# Scion Influence and Rootstock Specificity in Grafted Grapevines: Insights into Graft Affinity, Growth, and the Dynamics of Nutrient Acquisition

**DOI:** 10.3390/plants15182797

**Published:** 2026-09-12

**Authors:** Yonggang Yin, Changjiang Liu, Nan Jia, Xinyu Wang, Shuli Han, Yan Sun, Pengtao Shi, Shiyuan Liu, Qian Gao, Bin Han, Minmin Li

**Affiliations:** Changli Institute of Pomology, Hebei Academy of Agricultural and Forestry Sciences, Qinhuangdao 066600, China; yinyonggang00@hotmail.com (Y.Y.); nannanjia123@163.com (N.J.);

**Keywords:** mineral, element, *Vitis vinifera*, petiole, phenological stages

## Abstract

Grafting is a crucial strategy for improving stress resistance and fruit quality in grape production. However, our understanding of the specific roles of rootstocks in graft establishment, vine growth, and nutrient acquisition remains limited. This study evaluated the relevant traits of three worldwide wine grape cultivars grafted onto eight rootstocks, aiming to clarify the inherent characteristics of individual rootstocks and provide insights for their better understanding and utilization. The rootstock showed significant effects on petiole mineral levels with consistent patterns, even across developmental stages and years. For example, 5BB consistently accumulated Ca at all stages, while Beta demonstrated strong Mn uptake. Additionally, 101-14M and 110R specifically enriched B at the flowering stage. The scion showed a great influence on post-graft growth and mineral absorption. Specifically, ‘Cabernet Sauvignon’ (CS) exhibited superior performance in macronutrient accumulation, whereas ‘Petit Verdot’ (PV) showed distinct advantages in micronutrient. From flowering to veraison, the dominant mineral profile shifted from N, P, and B to Ca, Mg, and Cu, with the scion’s influence becoming evident at veraison. These findings enhance our understanding of rootstocks and provide a reference for their utilization.

## 1. Introduction

Breeders, horticulturists, and growers consistently seek cultivars that combine multiple desirable traits, including high yield, fruit quality, and stress tolerance [1,2]. However, integrating these characteristics into a single genotype is challenging in perennial fruit crops because of their complex genetic backgrounds and long breeding cycles. Grafting provides a horticultural strategy that can help alleviate this limitation. By combining genetically distinct rootstocks and scions, traits associated with the root system—such as tolerance to drought, soil-borne pests, mineral element deficiency, and unfavorable soil conditions—can be conferred to the aerial portion of the plant [3,4,5], thereby enabling the integration of multiple advantageous traits without genetic modification of the scion [6,7].

Rootstocks are essential in grape (*Vitis vinifera*) production. Their widespread use began with the control of phylloxera damage to European grapevines and has since expanded to mitigate various biotic and abiotic stresses [8]. The main goal of using rootstocks is to improve scion performance, with graft compatibility being a key requirement [9]. However, compatibility often varies across scion and rootstock genotypes, reflecting genetic differences and complex interactions between them [10,11]. Conclusions from different studies are often based on different evaluation criteria or frameworks. For example, evaluation methods vary significantly: parameters such as callus formation, budding, and rooting ability, and controlled experimental conditions are recognized as important factors in assessing graft compatibility, but they are only partially applied in various studies [12,13]. Additionally, subsequent graft responses, such as plant mortality or growth-related incompatibility, are critical indicators of graft compatibility [9,12]. These inconsistencies hinder direct comparisons among studies and obscure the intrinsic characteristics of rootstocks.

As the interface between plant and soil, rootstocks regulate the uptake and translocation of mineral elements to aboveground organs, thereby affecting nutrient balance and fertilization management [14,15]. Rootstocks exerted a stronger influence on the concentrations of most elements in grapevine petioles at veraison than either the scion cultivar or the experimental block, as revealed by a multi-grafting combination study, although the intrinsic nutrient uptake characteristics of individual rootstocks remain unclear [16]. In contrast, environmental factors such as growing season and vineyard location were shown to exert stronger effects on petiole nutrient concentrations of grafted Pinot Noir at anthesis than rootstock genotype, and vines grafted onto 3309C consistently exhibited P deficiency across three years [17]. Similarly, in the ‘BRS Vitória’ grapevine, year-to-year variation exerted broader effects than rootstock on the elemental composition of pruned canes after harvest.

In addition, variation in developmental stage and sampling tissue among studies, as mentioned above, may further contribute to inconsistent interpretations of rootstock-mediated nutrient uptake. Petioles are more commonly used to assess the nutritional status of grapevines [17,18,19]. Utilizing petioles allows for broader and more precise cross-study comparisons and discussions. The developmental stage represents another important factor when evaluating rootstock effects on mineral uptake, which was relatively under-explored. Grapevines undergo substantial physiological transitions during the growing season, and nutrient requirements vary markedly across phenological stages [20,21]. Comparisons without accounting for phenological stage may fail to reveal whether rootstocks exhibit specific patterns of nutrient regulation.

Theoretically, rootstocks should have inherent and potentially conserved effects on graft compatibility and mineral nutrient uptake in grafted plants, while these effects are also influenced by the scion genotype. Additionally, plant developmental processes and seasonal environmental conditions can impact this performance by acting on either the rootstock or the scion of the grafted plant. However, current independent studies provide limited evidence to support these patterns and often lack comparable datasets and evaluation frameworks that would allow for robust cross-study comparisons. Comprehensive assessments involving multiple rootstocks and different scion cultivars under consistent field conditions remain relatively rare, especially those that include graft performance, vegetative growth, and nutrient accumulation across key phenological stages.

Given that, we grafted three grapevine scion cultivars onto eight widely used rootstocks and evaluated them under the same vineyard conditions over a continuous experimental period. Indicators of graft performance, vegetative growth, and mineral element composition were analyzed, including petiole nutrient concentrations at the flowering and veraison stages. By integrating multiple physiological and nutritional traits through multivariate analysis, this study aims to (i) clarify the extent of rootstock influence on grafting compatibility and mineral uptake, (ii) characterize overall patterns of rootstock effects across different graft combinations, and (iii) evaluate whether specific rootstocks show consistent performance across scions and developmental stages. This study provides new insights into rootstock-mediated regulation of petiole nutrient status in grafted grapevines across developmental transitions, offering valuable implications for rootstock selection based on nutrient acquisition capacity.

## 2. Results

### 2.1. Meteorological and Soil Characteristics

**Figure 1:**
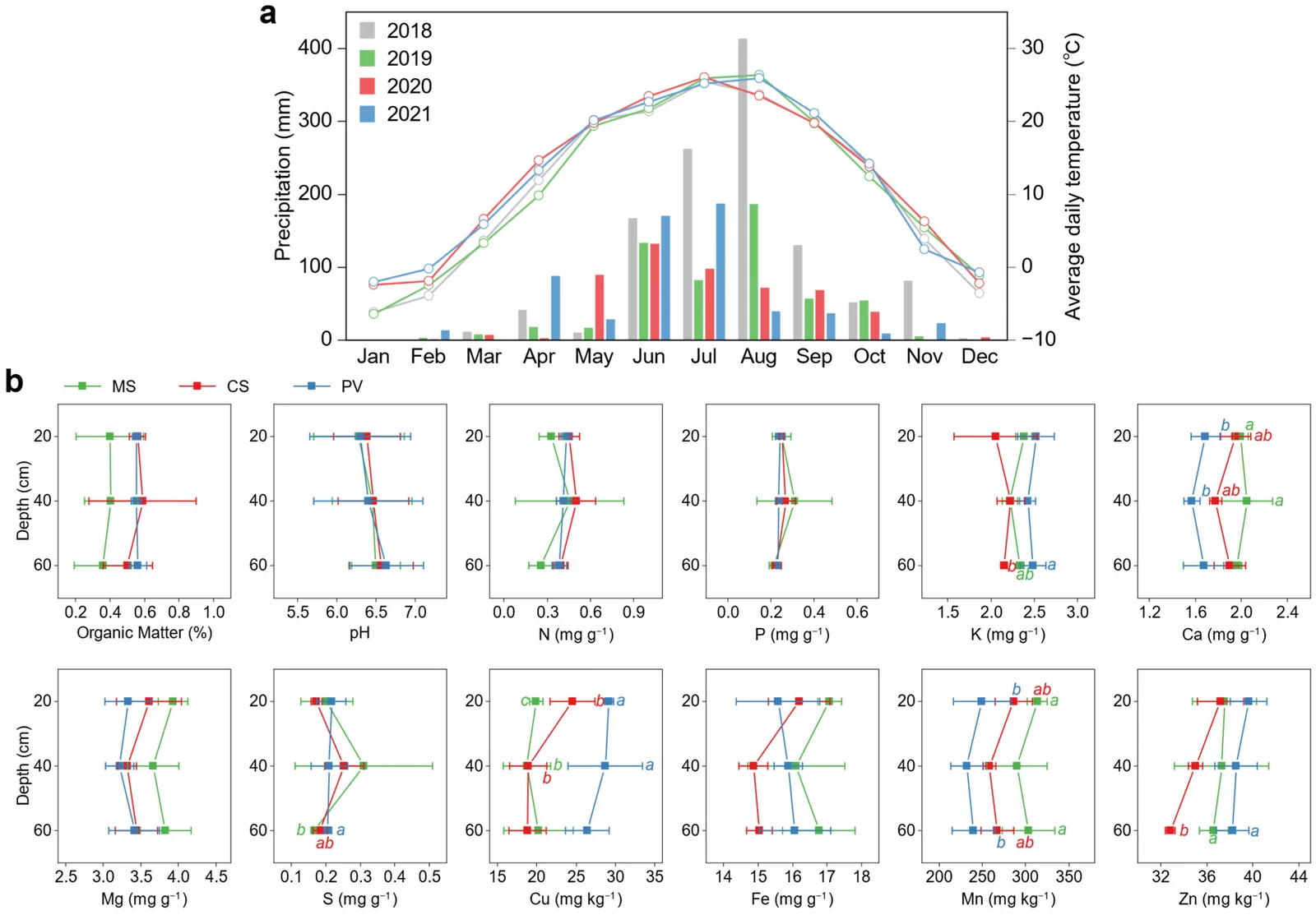
Analysis of the background data of the study. (**a**) meteorological data, where bars indicate precipitation and dots indicate temperatures, and (**b**) soil chemical profiles corresponding to each cultivar. CS, ‘Cabernet Sauvignon’; MS, ‘Marselan’; PV, ‘Petit Verdot’. Within each soil depth, different letters indicate significant differences (*p* < 0.05) according to Tukey’s post hoc test.

Seasonal trends in daily mean temperature were consistent across years (Figure 1a). The average temperature was 10.9 °C in 2018 and increased gradually in subsequent years (Table S2), with peak temperatures in July and August. Total yearly precipitation was significantly higher in 2018 (1174 mm) compared to 2019–2021, during which rainfall ranged from 515 to 598 mm, with the majority falling between June and August. Soil organic matter content was relatively low, spanning from 0.4% to 0.6% (Figure 1b). Soil pH varied from slightly acidic to neutral, between 6.0 and 7.0. No significant differences were observed in the levels of macronutrients N, P, K, and Mg among the planting areas of the three cultivars. In the 0–40 cm soil layer, ‘Petit Verdot’ (PV) planting areas exhibited lower Ca but higher Cu concentrations relative to ’Cabernet Sauvignon’ (CS) and ‘Marselan’ (MS) planting areas. Mn levels varied considerably overall and were relatively lower in the PV planting zone. Zinc levels in the CS planting zone decreased with increasing soil depth. Boron was not detected in the soil samples analyzed.

### 2.2. Multivariate Structure of Graft Establishment

#### 2.2.1. Effects of Scion and Rootstock on Graft Establishment

The scion genotype exerted dominant effects on graft healing index (GHI) and budbreak rate (Table 1 and Table S3), with $\eta_{p}^{2}$ values of 0.72 and 0.66, respectively (Figure S1a). PV exhibited the highest budbreak rate (78%), exceeding that of MS (57.8%) and CS (49.6%), and the highest GHI (0.65), exceeding that of MS (0.58) and CS (0.45). In contrast, rooting rate and callus forming index (CFI) were more influenced by rootstock genotype, with larger effect sizes of 0.67 ($\eta_{p}^{2}$) and 0.68 than the scion, respectively (Figure S1a). Grafts on 188-08 showed a rooting rate of 59.7%, followed by 101-14M (49.7%), both markedly higher than those on 5BB and SO_4_. Interaction effects of rootstock and scion on GHI, budbreak rate and CFI were also considerable, as indicated by the $\eta_{p}^{2}$ values near 0.5 (Figure S1a).

Rootstocks also significantly influenced shoot development, as evidenced by higher budbreak rates on SO_4_ (70.8%) and Beta (70%) than on 5BB (47%). Conversely, scion genotype also affected root initiation, although its effect was relatively small (Figure S1a); for example, MS grafts showed higher rooting rates than CS grafts. In addition, scion genotype influenced callus formation at both the graft interface (GHI) and the basal cutting surface (CFI). Specifically, both GHI and CFI showed a progressive decline from PV to MS and further to CS, indicating a strong scion-dependent gradient in callus development. By comparison, rootstock exerted a secondary but discernible effect, particularly on graft interface healing (GHI). Grafts on 101-14M and 5C developed substantially larger callus areas (with GHI 0.72 and 0.69, respectively) at the graft interface than those on 188-08 (0.35) and 110R (0.43). Across eight rootstocks, CFI values varied from 0.34 in 110R to 0.56 in 101-14M, whereas three scions displayed a range of 0.31–0.53.

Among all tested rootstocks, 101-14M showed both strong graft-healing capacity and high rooting efficiency, suggesting potential suitability for nursery production. In contrast, CS/5BB showed concurrent reductions in callus formation and rooting. Pronounced interaction effects were observed, including exceptionally high budbreak rates (>85%) in several PV combinations but severe suppression in CS/5BB and CS/188-08 (Table S3).

#### 2.2.2. Principal Component Analysis (PCA) on Graft Indicators

**Figure 2:**
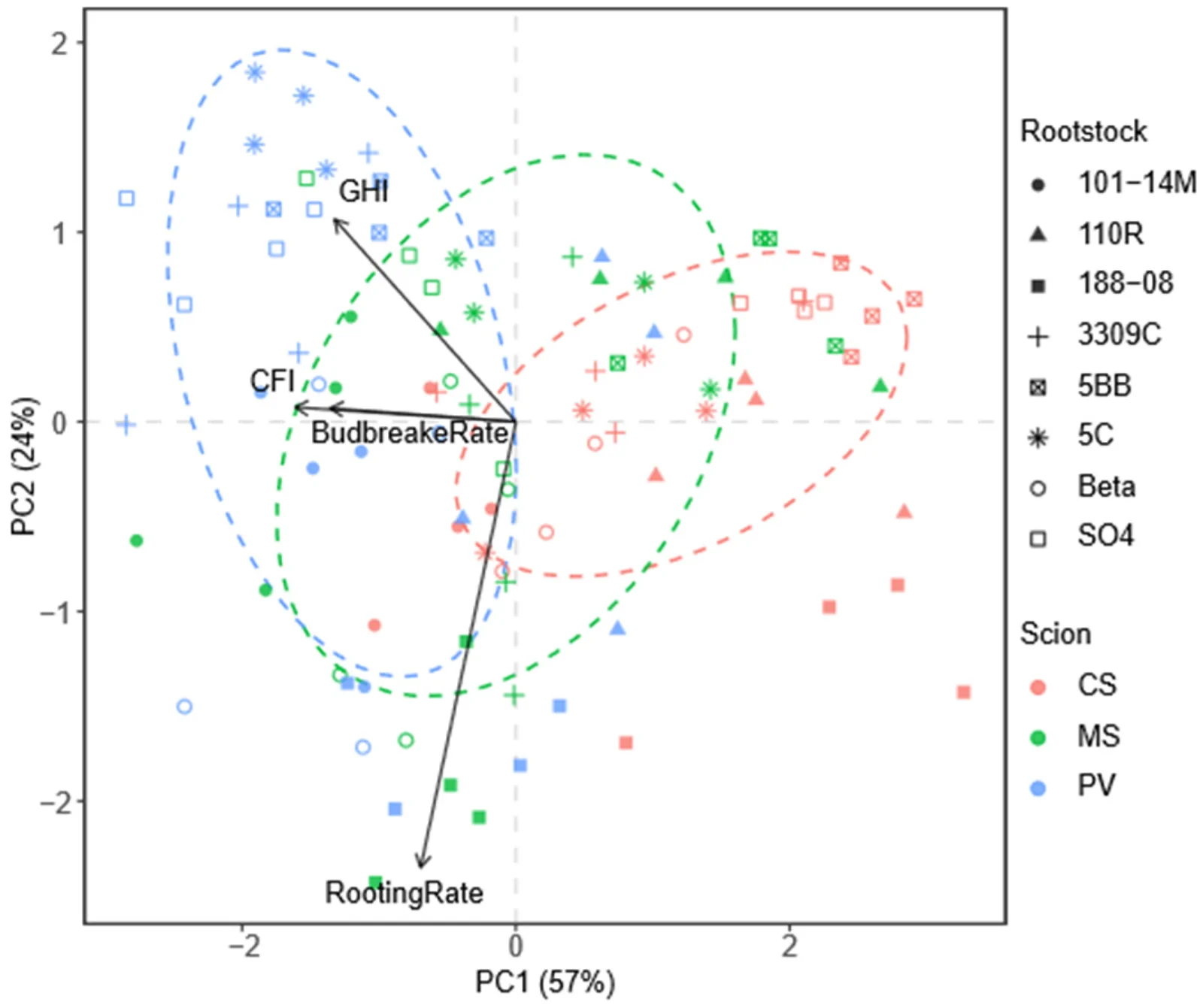
Biplot of PCA for grafting data. CFI indicates callus forming index, and GHI indicates graft healing index. Different scion cultivars are indicated by different colors, with red, green, and blue representing CS, MS, and PV, respectively. Dashed circles indicate 95% confidence ellipses, corresponding to each cultivar.

PCA explained the primary sources of variation in grafting performance, with the first two principal components explaining 81% of the total variance (PC1: 57%; PC2: 24%) (Figure 2). CFI and budbreak rate showed strong and nearly identical loadings on PC1, indicating a strong positive association between callus proliferation at the root interface and early shoot emergence. The graft healing index (GHI) showed a similar loading pattern, further reinforcing the linkage between tissue proliferation and initial scion growth. In contrast, rooting rate loaded primarily along PC2 and exhibited a clear separation from CFI, GHI, and budbreak. This orthogonal configuration suggested that root initiation represents an independent axis of graft performance, largely independent of callus formation, graft union quality, and shoot phenology.

### 2.3. Variation in Field Vegetative Growth Across Years and Scion–Rootstock Combinations

#### 2.3.1. Effects of Scion, Rootstock and Year on Early Vegetative Growth

Initial vegetative growth varied markedly among scion–rootstock combinations and between years (Table 2 and Table S4). Year effects, reflecting cumulative seasonal growth, together with scion genotype, were the primary determinants of early vine development, whereas rootstock effects were comparatively weaker and dependent on trait and year (Figure S1b). Effects of Interactions between scion and rootstock, and between scion and year, were also larger than the single effect of rootstock.

Survival rates were high (81–98%) after transplanting in the vineyard in 2018, indicating successful graft establishment from well-grafted plants, although overall vegetative growth remained limited during the establishment year. Beyond the pronounced year effects arising from cumulative growth across seasons, the scion exerted a larger influence than the rootstock on vine growth, as indicated by $\eta_{p}^{2}$ values (Figure S1b). For instance, CS-grafted plants exhibited a higher survival rate (93.8%) than PV (86.3%) and MS (85.2%), along with superior vegetative vigor in both scion and rootstock tissues and a greater affinity coefficient (AC = 1.3). In contrast, rootstock effects were relatively small. Among the rootstocks, grafts on 5BB, for example, exhibited the greatest rootstock diameter (16.5 mm), representing a 1.6 mm difference relative to the smallest diameter observed in Beta.

No significant rootstock influence was detected in PV, whereas in CS several growth-related traits were affected (Table S2). Rootstock influence was primarily reflected in lateral growth below the graft union. In CS grafts, combinations with 5BB, SO_4_, 101-14M, and 188-08 developed thicker stems below the graft union, while in MS grafts, such increases were observed mainly with 5BB (Table S4). Scion genotype thus emerged as a major determinant of scion–rootstock interaction effects.

Ungrafted vines displayed larger scion diameters than grafted vines during early growth. However, this difference progressively diminished and was eventually eliminated as grafted vines developed, except in CS grafted onto Beta. These patterns suggest progressive coordination between scion and rootstock during establishment, achieved either through rootstock-promoted scion growth (e.g., CS) or scion-constrained rootstock growth (e.g., MS). Nevertheless, certain rootstock–scion combinations appeared intrinsically less coordinated.

#### 2.3.2. PCA of Growth Parameters Across Two Years

**Figure 3:**
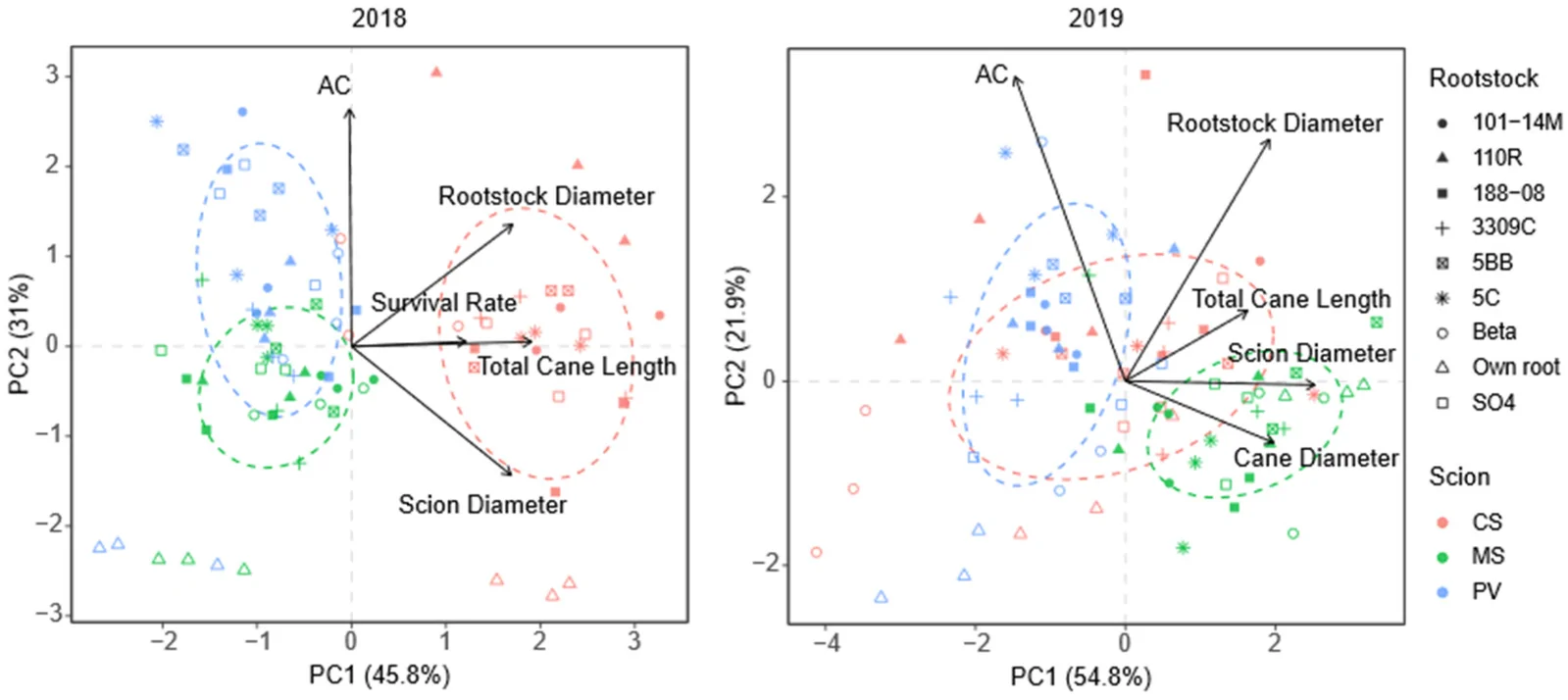
PCA plot based on growth data of three scion cultivars on eight rootstocks in 2018 and 2019. AC indicates the affinity coefficient. Different scion cultivars are indicated by different colors, with red, green, and blue representing CS, MS, and PV, respectively. Dashed circles indicate 95% confidence ellipses, corresponding to each cultivar.

The PCA results confirmed the pronounced effects of year and scion on vine growth (Figure 3). They further indicated that the survival rate in the planting year was positively associated with total cane length, while it showed moderate correlations with stem thickness of both the rootstock and the scion. No association with AC was detected. AC remained clearly separated from other variables in the following year, indicating its independence from growth-related traits. Overall, after vine establishment, marked biomass accumulation was observed in MS in the second year, followed by CS, and the biomass differences between scions progressively diminished.

### 2.4. Temporal and Genotype-Dependent Variation in Petiole Mineral Composition

Multifactorial analysis of variance showed that growth stage was the most significant source of variation for most elements, followed by year and scion, whereas the effect of rootstock was mostly minor (Table 3, Figure S1c). The interaction between developmental stage and year also showed considerable effect on some elements. The fundamental shift in the petiole nutrient profile from the florescence to veraison reflected the physiological transition from vegetative growth to reproductive maturation.

#### 2.4.1. Effects of Developmental Stage and Year on Mineral Uptake

**Figure 4:**
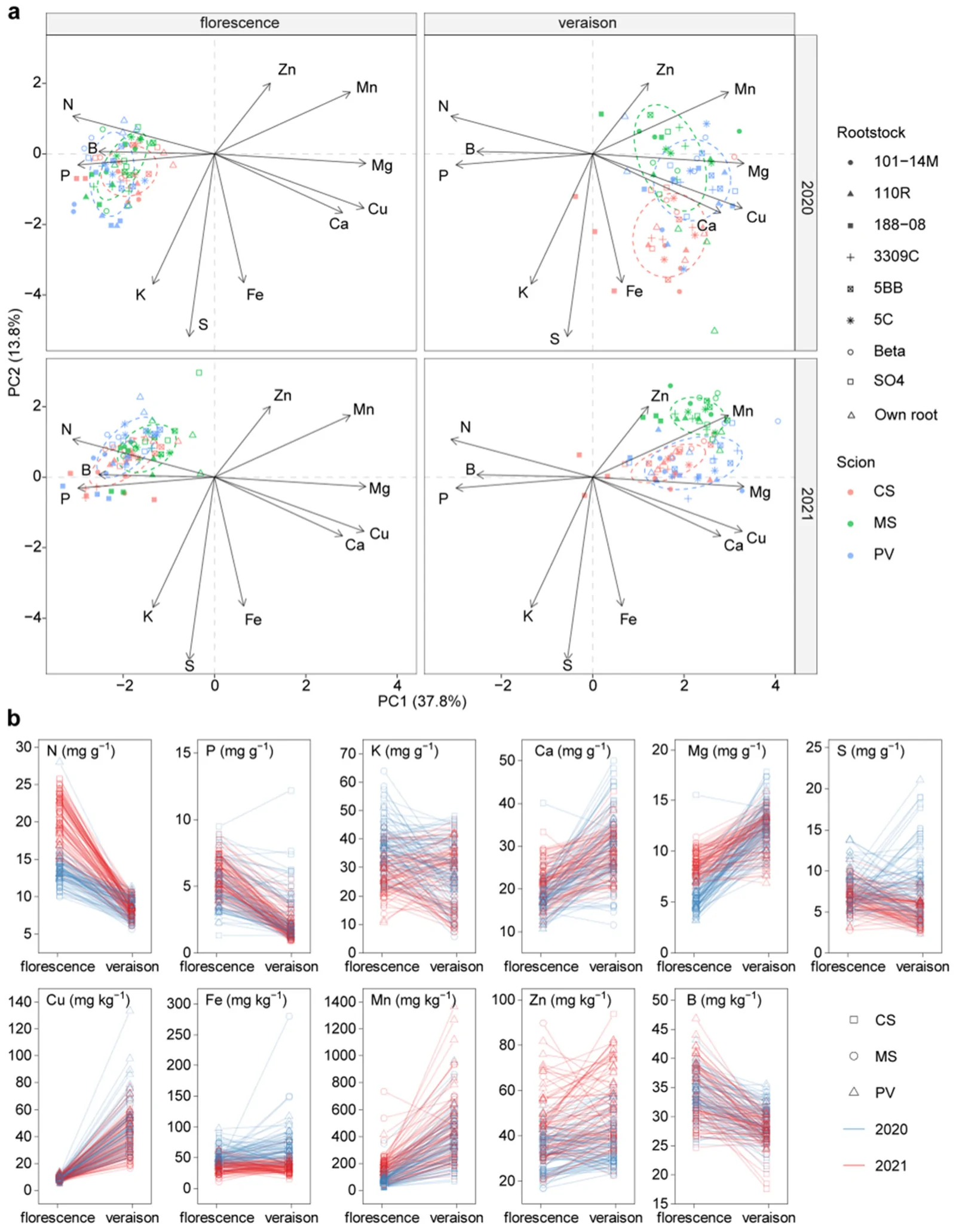
Petiole mineral nutrient profiles of three scion cultivars grafted onto eight rootstocks at two growth stages over two years. (**a**) PCA plots at the florescence and veraison stages of 2020 and 2021, with dashed circles indicating 95% confidence ellipses, corresponding to each cultivar; (**b**) differences in petiole nutrients between the two stages. Different scion cultivars are indicated by different colors, with red, green, and blue representing CS, MS, and PV, respectively.

PCA was conducted to evaluate the overall effects of multiple factors on petiole mineral composition (Figure 4a). PC1 and PC2 explained 37.8% and 13.8% of the total variance, clearly showing the main effects of developmental stage and year on nutrient uptake patterns. From flowering to veraison, vines underwent a distinct shift in nutrient uptake patterns, transitioning from a profile dominated by N, P, and B (negative side of PC1) to one characterized by greater accumulation of Ca, Cu, Mg, and Mn (positive side of PC1).

Macronutrients showed a marked decline as the season progressed (Table 3, Figure 4b). In total, N levels dropped 51%, from 17.2 mg g^−1^ at florescence to 8.4 mg g^−1^ at veraison; phosphorus decreased by 56%, and potassium declined 15% to 27.7 mg g^−1^. Conversely, calcium and magnesium concentrations increased 49% and 75% at veraison, respectively. Micronutrients Cu and Mn showed sharp increases of over four and two times at veraison, respectively. S remained stable during the two developmental stages, while B content declined. Fe and Zn also increased at the veraison stage, despite being more affected by the year.

Year, against this backdrop of substantial shift, exerted a clear influence on nutrient accumulation at both stages. Specifically, elemental profiles shifted from a higher uptake of S, K, and Fe in 2020, which mainly contributed to the negative side of PC2, toward increased accumulation of Zn, Mn, and N in 2021, which loaded more strongly on the positive side of PC2. Interannual variation also exerted strong effects on the uptake of certain mineral elements. N, Mn, and Zn levels in the petiole, for instance, were higher in 2021, while K, S, Fe, and Cu contents declined.

Notable interaction effects suggested that the magnitude of nutrient variation associated with the stage transition differed between years (Figure 4a). In 2021, N, B, and Mn exhibited greater shifts, whereas in 2020, Ca, Mg, and S showed more pronounced changes. Across samples, N concentrations declined at veraison, whereas Cu levels increased. These shifts were amplified in 2021 for N and in 2020 for Cu. In contrast, K, S, Fe, and Zn exhibited more variable and less uniform patterns across years and stages. Such differential responses reflect the complex interactions among rootstock, scion, stage, and year.

#### 2.4.2. Scion Effect on Accumulation of Macro- and Micronutrients

Scion, as an additional regulatory factor, exhibited a distinct preference in nutrient uptake. Variations in nutrient concentrations between scions became larger at veraison, mainly along the PC2 axis, encompassing Fe, S, K, Zn, and Mn (Figure 4a). CS showed a stronger association with the accumulation of macronutrients, including N, P, K, Ca, Mg, and S. Specifically, CS exhibited the highest overall mean concentrations of the macronutrients N, P, K, Ca, and Mg, reaching 13.4, 4.9, 36.1, 27.5, and 10.7 mg g^−1^, respectively (Table 3). PV was more closely associated with the uptake of N, S, and several micronutrients, including B, Cu, Fe, Mn, and Zn, while maintaining relatively high K uptake. The mean concentrations of B, Cu, Fe, Mn, and Zn for PV reached 33.6, 33.9, 50.5, 318.4, and 52 mg kg^−1^, respectively. In contrast, MS generally exhibited intermediate to relatively lower element concentrations (Table 3).

The coefficients of variation for elemental concentrations were generally larger among scions than among rootstocks, with the notable exceptions of Mn and Ca (Table 3). This increased variability of Mn and Ca among rootstocks was largely attributable to Beta, which exhibited markedly high Mn accumulation while maintaining substantially lower Ca concentrations.

#### 2.4.3. Rootstock-Specific Effects on Nutrient Uptake

Although rootstock significantly influenced nutrient uptake, its effects were partially masked by stronger sources of variation (Table 3, Figure S1c). Overall, grafted vines exhibited enhanced uptake of N, P, and K compared with own-rooted vines, except for those on 101-14M and 110R, in which P uptake was lower than that observed in own-rooted vines. Vines grafted onto 188-08 showed lower uptake of Ca and Mg compared with those on other rootstocks. In contrast, vines on 101-14M, 110R, and 188-08 exhibited relatively higher S accumulation, while B concentrations were comparable to those observed in own-rooted vines. In addition, vines grafted onto 5BB displayed lower B levels. Neither grafting status nor rootstock choice resulted in marked differences in Cu and Fe concentrations (Table 3).

Hierarchical clustering of elemental profiles primarily grouped samples by scion genotype, particularly at veraison, when rootstock-related patterns were difficult to discern (Figure S2). To minimize the influence of inter-elemental differences in magnitude on clustering results, the concentration of each element was standardized across all samples using Z-score normalization before clustering analysis. This normalization revealed clearer rootstock-associated patterns.

**Figure 5:**
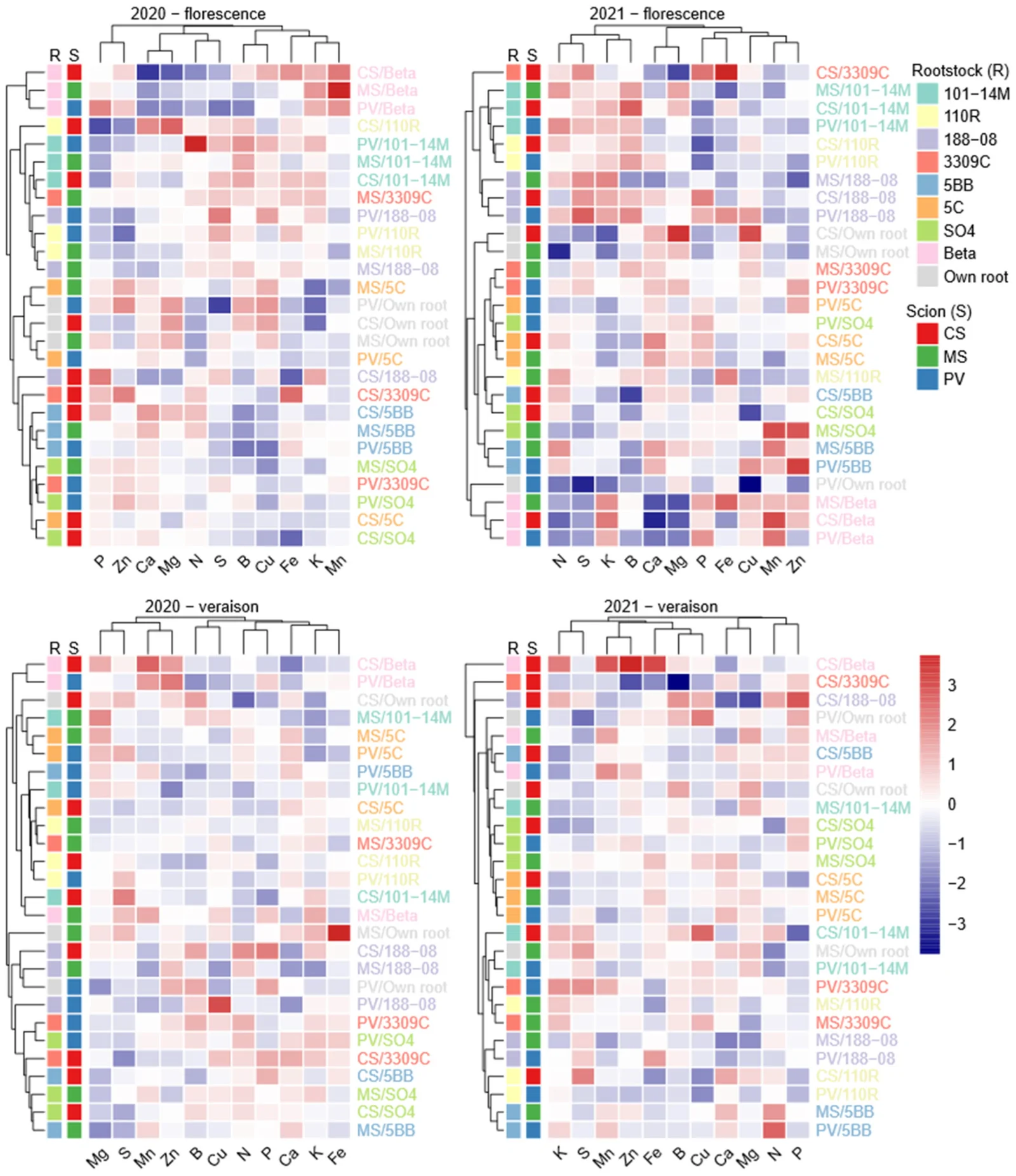
Characteristics of rootstock effects on mineral uptake as indicated with clustered heatmaps at floresence and veraison stages across two years. The color block in each heatmap indicates the z-score of each sample derived from each nutrient element.

At flowering, rootstock effects were already evident, although own-rooted vines showed relatively similar nutrient profiles among the three scion genotypes (Figure 5). Beta exhibited a distinct nutrient accumulation pattern characterized by higher Mn, K, P, and Zn levels but lower Ca, Mg, N, and S concentrations. In contrast, 101-14M, 110R, and 188-08 showed enhanced accumulation of S, K, B, and N, although some differences in Cu and P contributed to their separation. Notably, 5BB displayed a nutrient profile opposite to that of 101-14M, particularly for K, S, and B accumulation, a pattern that was also maintained at later developmental stages. At veraison, scion–rootstock interactions became more pronounced (Figure 5), with several rootstocks showing relatively conserved nutrient acquisition patterns across graft combinations. Beta-grafted vines consistently favored Mn accumulation but showed limited Ca accumulation, whereas 101-14M was characterized by reduced Ca and Mn uptake but relatively enhanced Mg accumulation. Vines grafted on 3309C exhibited lower Mg but higher K and Cu levels. Overall, although scion–rootstock interactions (Figures S3–S6) introduced considerable variations in specific graft combinations, rootstock-associated nutrient acquisition patterns remained detectable across developmental stages.

### 2.5. Element Interactions and Their Links to Graft Compatibility and Early Growth

**Figure 6:**
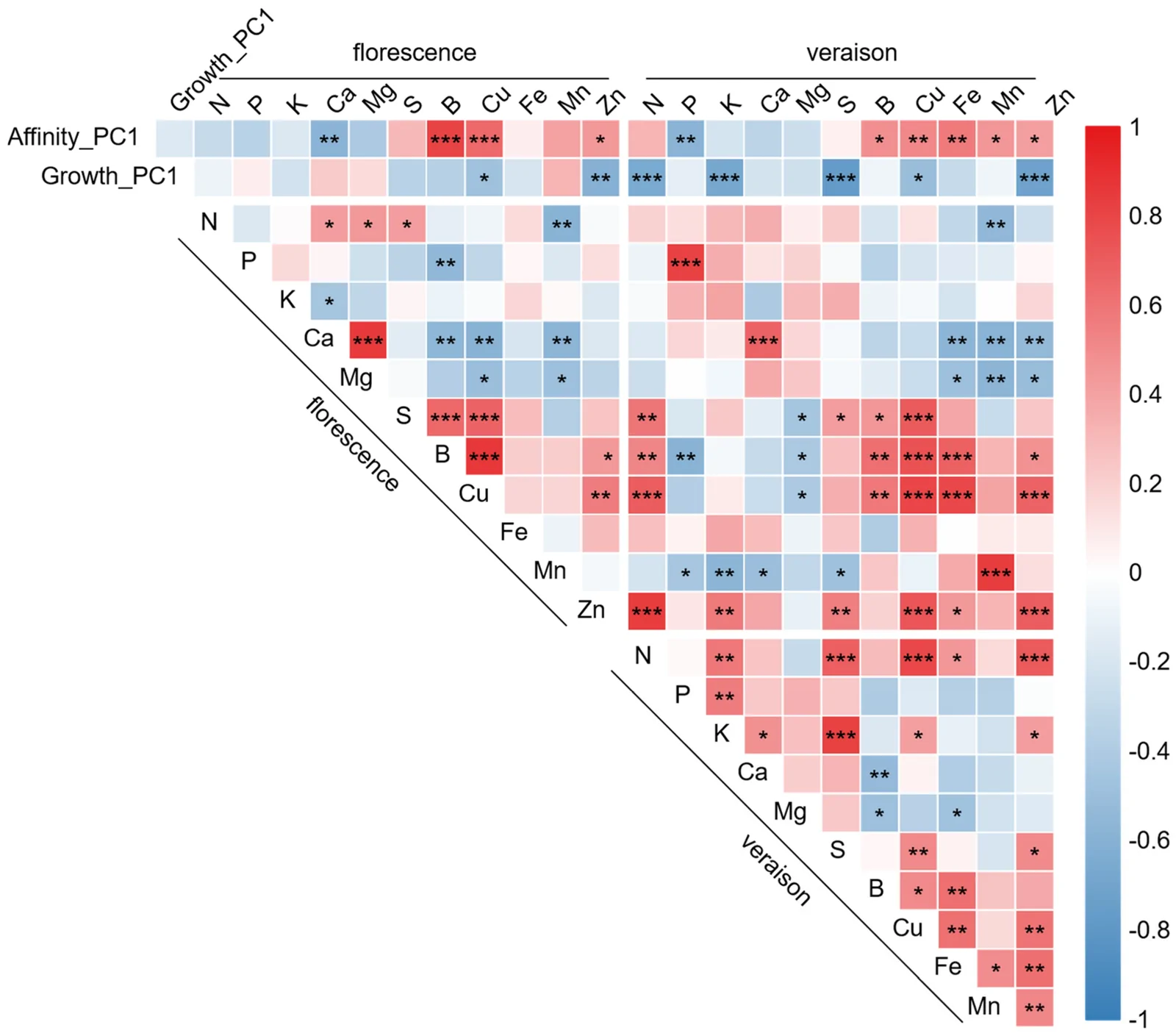
Correlation heatmaps of graft affinity, growth, and petiole mineral levels across two stages. *, **, and *** indicate *p* < 0.05, *p* < 0.01, and *p* < 0.001, respectively.

Stage-specific positive and negative correlations were observed among mineral elements (Figure 6, Table S5). At flowering, Ca was positively correlated with Mg (r = 0.85) but negatively correlated with B, Cu, and Mn, whereas Cu, B, and S showed significant positive intercorrelations. At veraison, correlations among mineral elements became more tightly coordinated. Strong positive correlations (r > 0.8) were observed between N and Cu, and between K and S. In addition, relatively high correlation coefficients were observed among N/P and K, N and S, B/Cu and Fe, and between Zn and Cu, Fe, and Mn. Notable correlations were also observed between the flowering and veraison stages for petiole P, Ca, Cu, Mn, and Zn concentrations (r = 0.68–0.84).

PC1 derived from affinity indices was strongly correlated with petiole B concentration at flowering (r = 0.81). It was also positively associated with Cu concentrations at both flowering (r = 0.65) and veraison (r = 0.52). In contrast, PC1 derived from growth indices was significantly negatively correlated with N, K, S, and Zn concentrations at veraison (−0.65 < r < −0.76).

## 3. Discussion

### 3.1. The Influence of Scion on Graft Compatibility, Growth, and Mineral Uptake

Although numerous studies have investigated the effects of rootstocks on scion growth and nutritional status [17,22], few studies have focused on the influences exerted by the scion itself. The present study showed that the scion had a larger effect than the rootstock on some attributes related to graft compatibility, growth vigor, and petiole mineral element concentration.

During the graft-nursery stage, root formation is linked to the rootstock genotype, and bud break is largely determined by the scion genotype (Table 1), consistent with previous studies’ observations [23]. The rootstock also influenced callus formation on both the lower portion of the rootstock and the graft union within each scion cultivar (Table S3), as seen in graft studies on single scion cultivars like ‘Red Globe’ and ‘Pusa Urvashi’ [11,24]. Daler et al. [23] reported that neither the rootstock nor the scion significantly affected callus development in Ω-type grafting of ten local cultivars onto four commercial rootstocks in Turkey, although rootstocks had a stronger effect than scions on most growth indices of the grafted saplings. Cookson et al. suggested that graft union formation happens at the same time as cambial reactivation in dormant cuttings during spring. Differences in the timing or strength of cambial reactivation among genotypes may therefore lead to variations in graft compatibility [25,26]. Auxin is widely seen as a key factor in callus formation during graft healing [27]. In hardwood grafts, actively growing scion tissues are considered the main sources of auxin, which promotes callus proliferation and vascular reconnection at the graft union. Once vascular continuity is established, auxin can be transported basipetally into the rootstock, potentially stimulating basal callus formation [28]. In the present study, a significant correlation (r = 0.7) was observed between GHI and CFI (Figure S7), which may partly reflect the hormone transport process described above, but further verification is needed through direct measurements of endogenous hormone levels.

The scion also had major effects on growth indicators, consistent with previous findings from biomass evaluations of five-month-old grafted vines [29]. A similar trend was also observed in perennial grapevines. In a vineyard established on heavy clay soils, the scion cultivar not only exerted primary control over the aboveground growth of grafted vines but also had crucial effects on root development and distribution [30]. The great effect of the scion on shoot growth primarily reflects its control over canopy development and carbon assimilation.

Scions were reported to have a stronger impact than rootstocks on N, K, and Ca concentrations in petioles during the veraison stage, but the influence of rootstock was more significant in most instances [19]. This discrepancy could be attributed to the number of grafting combinations. In the previous study, four scion cultivars were grafted onto 55 rootstocks across seven genetic backgrounds; this scale far exceeds ours and may involve broader genetic variation. When two scion cultivars were grafted onto four rootstocks under hyper-arid conditions, the effects of scion and rootstock were nutrient-dependent [31]. To clarify whether the notable effect of the scion on petiole elemental composition observed in this study was incidental, we performed a regression analysis between the mean elemental concentrations of each scion type in grafted vines and those measured in ungrafted vines, which represent the intrinsic characteristics of the scion. The results showed a strong concordance between the two (Figure S8), indicating that even under grafted conditions, the scion largely retains its intrinsic characteristics in regulating nutrient uptake. This is rarely discussed in previous studies because few ungrafted varieties are included in multi-cultivar studies.

### 3.2. Subtle Yet Distinctive Effects of Rootstock on Nutrient Uptake

The rootstocks significantly influenced the mineral uptake within each scion cultivar, as observed in grafting studies involving other cultivars [8]. The elevated P and K accumulation in Beta aligns with previous evidence of its efficient P and K utilization [32]. Higher K accumulation may contribute to its strong cold tolerance [33] by supporting osmotic adjustment, cellular water balance, and membrane stability. P availability has been directly associated with vegetative growth in grapevine, which may contribute to the vigorous growth phenotype of Beta [34]. High levels of Mn accumulation observed in Beta may lead to Mn toxicity risk according to sufficiency ranges referenced [19,35]. The extensive root system of Beta may facilitate the uptake of deep-soil available Mn, or its roots may possess a strong capacity for proton secretion to increase soil acidity [36]. Likewise, a surge in petiole Mn levels in own-rooted Cabernet Sauvignon and Chardonnay at veraison has been reported, corroborating the intense demand for Mn during this developmental stage [37]. On the other hand, higher Mn enrichment may be accompanied by a risk of Ca and Mg deficiencies because of ion antagonism. Unchanged petiole S contents between stages were also revealed [37,38]. The pronounced B accumulation in 101-14M and 110R during flowering mirrors patterns seen in ‘Chambourcin’ and ‘Cabernet Sauvignon’ grafting studies [39,40], while no such advantage was found in ‘Pinot Noir’ [17]. The root systems of 101-14M and 110R differ significantly in their architecture: the former is characterized as a shallow-rooted genotype, whereas the latter exhibits a deep-rooted pattern [41]. Since available B is predominantly enriched in the topsoil in the form of borate ions, this spatial distribution may explain the preferential B uptake observed in 101-14M. In contrast, the B accumulation in 110R may be attributed to its highly efficient water uptake capacity [42], which facilitates the mass flow of B towards the root surface. 5BB proved to be an efficient Ca accumulator across almost all phenological stages, and it effectively increased the leaf Ca content of the ‘Ráthay’ grape as revealed in a long-term field trial [43]. The genetic background of the rootstock was considered crucial in shaping petiole mineral status [19]. 5C and SO_4_, which share the same *V. berlandieri* × *V. riparia* hybrid origin as 5BB, exhibited similar Ca-uptake patterns. However, significant variations exist even within the same genetic background; for instance, 5C consistently exhibited weaker K uptake compared to other rootstocks. 5BB is regarded as an excellent lime-tolerant rootstock [44], and its adaptation to high-calcium environments may have driven the evolution of its intracellular calcium sequestration mechanisms [45]. Given the critical importance of potassium for both fruit yield and quality [46], caution is advised when grafting onto rootstock 5C in K-deficient soils. On the other hand, even scions sharing the same *V. vinifera* genetic background exert a noticeable influence on petiole mineral status.

### 3.3. Phenological Stage Reshapes Vine Petiole Mineral Profiles

These rootstock effects are also predicated on a broader context in which vines’ mineral nutrient demands vary markedly across phenological stages [38]. The rootstock effect was more readily discernible at flowering (Figure 5), but less notable as the season progressed to veraison, when the effect of the scion genotype intensified. Similarly, Baby et al. (2021) reported that variability in petiole mineral levels among ungrafted grape cultivars increases at veraison [37].

The decrease in N, P, and B from flowering to veraison reflected the slowdown in vegetative growth of these basal leaves and the reallocation of resources to berry development. This distinct flow of macronutrients from source to sink (fruit) was monitored during the growing season [47]. Similar declines in petiole N and P concentrations have been documented in prior studies on ‘Thompson Seedless’ [48] and ‘Tempranillo’ [49]. The surge in Cu, Mn, Mg, and Ca at veraison was also observed in leaves of ‘Pinot Noir’ [50] and ‘Tempranillo’ [49]. Investigations into the nutrient dynamics of ‘Merlot’ grapevine across three developmental stages showed consistent fluctuations in most petiole minerals, and Ca, Mg, Mn, and Zn were further enriched in the petiole as the maturation stage advanced [51]. Notably, these elements tend to accumulate more in the petiole and leaf blade than in the fruit. Petioles, like stems, may store and release nutrients [37,52]. Therefore, the surge in these minerals likely reflects that, as leaves expand and reach maturity, their demand for N and P decreases sharply; instead, they enhance photosynthesis to support the carbon needs of rapid fruit development.

### 3.4. Interactions Among Mineral Elements

Coordinated homeostasis between Ca and Mg results from their joint contributions to plant cell structural stability and enzymatic activity [53]. Similarly, the synergistic relationship between N and S is attributed to N–S signaling crosstalk, which plays a critical role in maintaining nutrient homeostasis and balanced plant growth [54]. The Ca–Mn antagonism observed in the present study may be attributed to competitive interactions between these two divalent cations during root surface adsorption, apoplastic exchange, and membrane transport processes [55]. Competition among chemically similar ions, including K^+^, Ca^2+^, Mg^2+^, and Mn^2+^, may occur during membrane transport, through interactions with shared transport systems, and in osmotic regulation [56,57], contributing to the nutrient trade-offs observed among different rootstocks. These effects were particularly pronounced in rootstocks with preferential accumulation of specific ions, such as Beta for Mn and 5BB for Ca [57]. Although Ca and B generally exhibit coordinated roles in maintaining cell wall integrity [58], excessive Ca accumulation may alter B availability through changes in cell wall binding and nutrient balance. The relationship between Ca and B is genotype-dependent. The occurrence of antagonism may be associated with excessive accumulation of certain ions, leading to imbalances in nutrient homeostasis.

### 3.5. Environmental Factors Further Complicate Rootstock and Scion Effects

Variability among years encompasses changes in meteorology, soil conditions, and other unknown factors, which can affect the growth and adaptability of rootstocks and scions, including their nutrient uptake performance [19]. In the present study, interannual environmental differences driven by precipitation may be linked to shifts in the accumulation of certain elements in petioles. Specifically, the year with higher rainfall showed enhanced uptake of N, Zn, and Mn (Table 3 and Table S2). However, this association is questionable. While rainfall may enhance nutrient availability, it can also lead to nutrient leaching. Soil physicochemical properties directly affect mineral uptake in grapevines [59]. However, the soil sampling design was not paired with the sampling for elemental determination, and soil nutrient levels were not treated as covariates in the analysis of variance. Therefore, the effects of soil status on nutrient acquisition cannot be quantified. Despite the soil properties from planting areas of three cultivars being comparable, the observed disparities in petiole elemental composition—specifically Ca and Cu—may be attributed to the variations in their respective soil availabilities. However, a discrepancy was noted for Mn, where soil content failed to predict the elemental concentration in the plants. In addition, the correlation analysis is further complicated by the fact that total soil elemental concentrations were used, rather than the bioavailable fractions.

It is noteworthy that the PC1 derived from growth-related parameters showed negative correlations with several nutrient elements. This suggests that biomass accumulation may contribute to a dilution effect on nutrient concentrations among graft combinations. Although the measurements of growth parameters and nutrient concentrations were conducted at different time points and may have been affected by varying environmental conditions, growth traits can still reflect the inherent vigor characteristics associated with different genotypes to some extent.

## 4. Materials and Methods

### 4.1. Plant Materials and Grafting Details

Three scion cultivars, ‘Cabernet Sauvignon’ (CS), ‘Marselan’ (MS), and ‘Petit Verdot’ (PV) were grafted on eight rootstocks, including ‘101-14M’, ‘110R’, ‘188-08’, 3309C’, ‘5BB’, ‘5C’, ‘SO_4_’ and ‘Beta’ (details of genotypes are presented in Table S1). The scion cultivars were obtained from the National Jujube and Grape Germplasm Repository (Taigu, Shanxi) in 2011, and all the rootstock cultivars were obtained from the National Grape and Peach Germplasm Repository (Zhengzhou, Henan) in 2008. All the introduced accessions are preserved in the Kongzhuang experimental station of Changli Institute of Pomology, Hebei Academy of Agriculture and Forestry Sciences. The entire experimental program covered a five-year period, consisting of grafting assessments in 2017 followed by field experiments conducted from 2018 to 2021.

Healthy one-year-old canes were harvested from rootstock and scion plants before the winter of 2016 and stored underground at approximately 60 cm depth until used for grafting in the spring of 2017. Hardwood cleft grafting was employed as described previously [11]. Briefly, scion cuttings with one intact bud were whittled into a wedge and inserted into the rootstock cuttings. The rootstocks were split in the middle, had their buds removed, and had their basal ends cut at a 30° angle. The graft union was sealed with grafting tape. The upper end of the scion was quickly dipped in wax, and the basal end of the rootstock was dipped into a rooting solution for 1 min. The rooting solution was prepared by dissolving rooting powder containing 20% naphthylacetic acid (NAA) and 30% indole-3-acetic acid (IAA) (Aibidi Biotechnology, Beijing, China) in anhydrous ethanol, followed by a final 5000-fold dilution with distilled water. The grafts were bundled in groups of ten and placed on a bottom-heated sand bed maintained at 25 °C and 75% humidity for 32 days.

### 4.2. Evaluation of Grafting Compatibility

Each replicate consisted of one bundle containing 10 grafts, and four bundles were randomly selected for each graft combination for evaluation. A previous evaluation system, including budbreak rate, rooting rate, callus-forming index (CFI), and graft-healing index (GHI), was adopted [11]. Budbreak rate or rooting rate was the percentage of grafts showing visible green tissue or roots. The grade of callus formation on the basal cut surface (*i*) or the graft cross-section (*j*) was based on the percentage of callus coverage area, which is visually estimated as follows: 0, 0%; 1, 1–25%; 2, 26–50%; 3, 51–75%; and 4, 76–100%.

CFI = (*i* × N*_i_*)/(4 × 10), (1)

where N*_i_* represents the number of cuttings exhibiting the corresponding callus-forming grade *i* (*i* = 0, 1, …, 4).

GHI = (*j* × N*_j_*)/(4 × 10), (2)

where N*_j_* represents the number of cuttings exhibiting the corresponding graft healing grade *j* (*j* = 0, 1, …, 4).

### 4.3. Vine Management, Experimental Design, and Post-Graft Growth Evaluation

**Figure 7:**
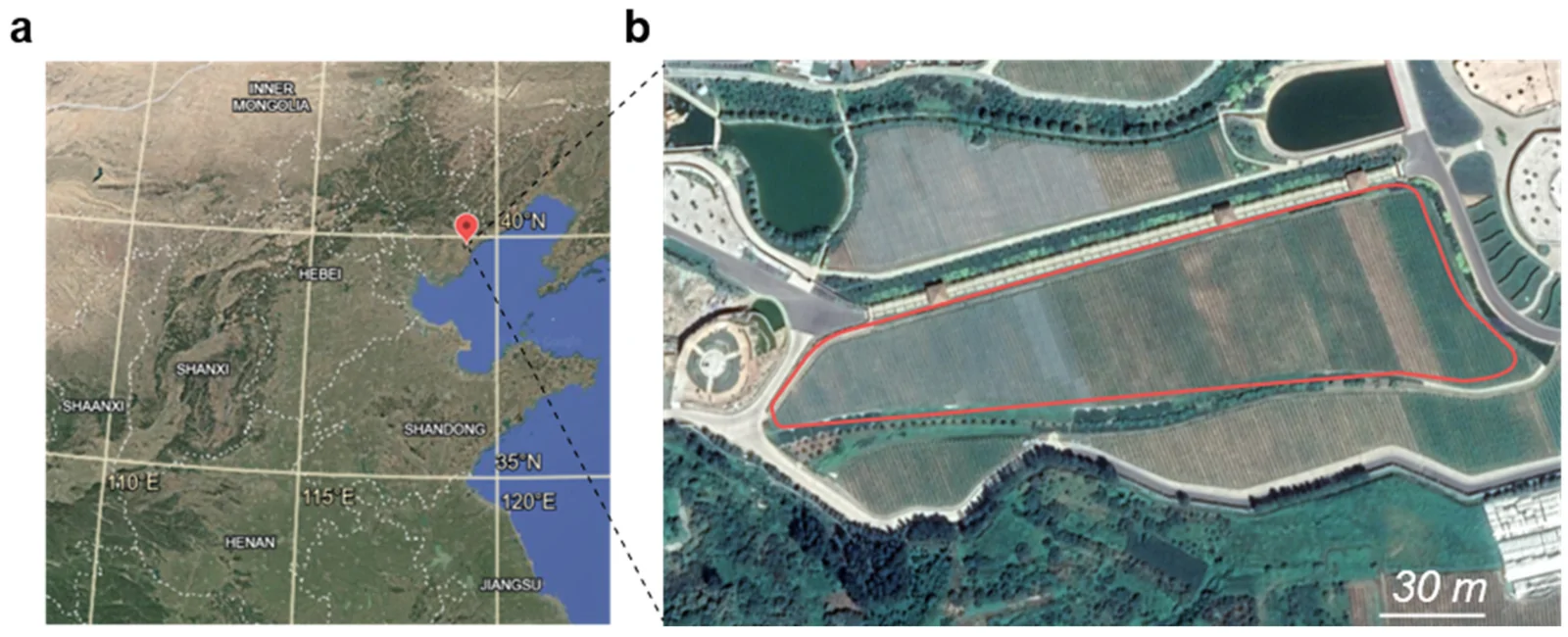
Experimental site. (**a**) Location of the winery and, (**b**) the overview of the experimental site.

Well-grafted and ungrafted vines of CS, MS, and PV were planted in Jinshi Winery in 2018. Before planting, the vineyard field underwent deep ploughing and land leveling. This vineyard is located in Changli, a major wine-producing region in China (Figure 7). The soil is typically sandy loam. Vines were trained to a vertical shoot positioning (VSP) system, with vine spacing of 0.8 m and row spacing of 2.5 m. One-bud spur pruning was performed after the leaves fell. A drip irrigation system was applied.

A randomized complete block design was employed. Three replicate plots were applied. Nine treatments, including eight graft combinations and one ungrafted control, for each cultivar, were arranged by row and randomly assigned within each plot. Each row comprised more than 25 vines.

Before winter pruning, trunk diameters below and above the graft union were measured using digital vernier calipers and recorded as rootstock and scion diameters, respectively. The affinity coefficient (AC) was defined as the ratio of rootstock diameter to scion diameter. Rootstock diameter was not measured for own-rooted vines since no rootstock was used, but scion diameter was measured at around 10 cm above the ground (approximately at the graft union of those grafted vines). Total cane length was calculated as the sum of all annual cane lengths. Survival rate was assessed in the first year, and cane diameter was measured in the second year. For each year, ten vines were randomly selected from each replicate row and considered as one replicate for trait measurement.

### 4.4. Total Mineral Nutrition Determination

Petioles opposite the inflorescences/clusters were sampled at flowering (50% caps off) and veraison (50% berries coloring) across two consecutive years. At each phenological stage in each year, 15 vines were randomly selected from each replicate, and one petiole was collected per vine, yielding 15 petioles per replicate. Petioles were washed twice with deionized water. After removing surface moisture with filter paper, petioles in paper bags were oven-dried at 60 °C to constant weight. Dried petioles were ground into powder in a grinding jar. For phosphorus (P), potassium (K), calcium (Ca), magnesium (Mg), sulfur (S), manganese (Mn), zinc (Zn), iron (Fe), boron (B), and copper (Cu) analysis, around 0.2 g of sample was digested with 5 mL nitric acid in a Mars 6 microwave digester (CEM, Mattews, NC, USA). Approximately 1 mL of the digested solution was filtered and made up to 50 mL with deionized water. Mineral nutrient contents were measured using an ICP-OES system (Optima 8000, PerkinElmer, USA) based on the standard curve constructed from standards. Nitrogen (N) content was analyzed using the Kjeldahl method [60]. Briefly, around 0.5 g of the samples were digested with H_2_SO_4_ and CuSO_4_, and the digested solution was distilled with excess NaOH. The released ammonia was absorbed by boric acid to form ammonium borate, which was subsequently titrated with HCl to determine the total nitrogen content.

### 4.5. Analysis of Soil Chemical Properties

In the spring of 2021, Soil samples were collected in late autumn from three soil depths: 0–20 cm, 20–40 cm, and 40–60 cm. In each plot, for each scion cultivar, six sampling sites 40 cm from the basal trunk were randomly selected. Soil samples from the same depth were air-dried, ground into a fine powder, and thoroughly mixed to form a composite replicate sample for each scion cultivar. The determination of soil mineral nutrition was conducted as described above. The soil organic matter (SOM) content was determined using the potassium dichromate (K_2_Cr_2_O_7_) oxidation method with external heating, as described by Nelson and Sommers [61]. Soil pH was measured in a 1:2.5 (*w*/*v*) soil-to-water suspension using a digital pH meter (TitroLine easy, Schott Instruments, Germany) after 30 min of equilibrium [62]. The electrode was calibrated using standard buffer solutions at pH 4.0, 7.0, and 10.0 before measurement.

### 4.6. Statistical Analysis

The primary data were organized and managed using Excel 2021 (Microsoft Corp., Redmond, WA, USA). Statistical analyses were conducted in SPSS 21 (IBM Corp., Armonk, NY, USA). Analysis of variance (ANOVA) was performed to evaluate differences among factors, following verification of normality (Shapiro–Wilk test) and homogeneity of variance (Levene’s test). Depending on the number of experimental factors involved, multi-way ANOVA models were applied. Effect-size comparison among factors was based on $\eta_{p}^{2}$, i.e., the sum of squares for the factor divided by the sum of squares for the factor plus the residual sum of squares. Year, phenological stage, scion genotype, and rootstock system were considered fixed factors in the corresponding ANOVA models, and all biologically relevant interaction terms were included. All the scion and rootstock combinations from each growth stage and year were subjected to a one-way ANOVA to reveal specific interactions between scion and rootstock. When significant effects were detected, means were compared using Tukey’s post hoc test at *p* < 0.05.

Principal component analysis (PCA), heatmap visualization, and correlation analysis were performed using RStudio 2026.01.0 (Posit Software, PBC, Boston, MA, USA) based on R version 4.5.2 [63]. Before PCA, the data were centered and scaled to unit variance (z-score standardization) to eliminate the influence of differences in measurement units and variable magnitudes. Heatmaps were generated from both original data and column-wise z-score standardized data to facilitate comparisons among treatments. Pearson correlation analyses were conducted using the mean data. The packages *ggplot2* [64], *pheatmap* [65], and *corrplot* [66] were used for data visualization and correlation analysis. Linear regression analyses and additional graphical visualizations were generated using OriginPro 2024 (OriginLab Corp., Northampton, MA, USA).

## 5. Conclusions

Our results have revealed the combined effects of scion genotype, rootstock characteristics, developmental stage, and environmental conditions on nutrient acquisition. The scion exerts a greater influence than the rootstock system on growth performance and mineral concentration. Rootstocks contribute substantially to graft establishment and markedly influence petiole Ca and Mn levels. Across years and phenological stages, Beta exhibited high Mn-uptake capacity yet lower Ca acquisition, whereas 5BB showed a preference for Ca uptake. Strong B accumulation was observed for 101-14M and 110R at the flowering stage. The strength of the rootstock/scion effects on nutrient acquisition varies between the developmental stages; specifically, differences among scion varieties became notably distinct at veraison, especially in the accumulation of S, K, Fe, and Zn. The shift from flowering to veraison altered the mineral uptake pattern, changing the focus from N, P, and B to Ca, Mg, Mn, and Cu. These findings provide further insights into the effects of rootstock on vine growth and nutrient acquisition, while also highlighting the importance of considering developmental stages when evaluating vine nutritional status. Despite this, we must acknowledge that varietal differences and limited sample sizes may contribute to these findings. Considering the critical role of environmental variables, such as climate and soil, validation across diverse habitats is necessary to strengthen the robustness of our conclusions.

## Figures and Tables

**Table 1:** Grafting-related indices of three grapevine cultivars grafted onto eight rootstocks.

Factor	Subfactor	CFI	GHI	Budbreak Rate (%)	Rooting Rate (%)
Scion	CS	0.31 ± 0.11 c	0.45 ± 0.15 c	49.6 ± 15.7 c	24.0 ± 19.0 b
	MS	0.45 ± 0.12 b	0.58 ± 0.14 b	57.8 ± 16.2 b	33.4 ± 27.8 a
	PV	0.53 ± 0.10 a	0.65 ± 0.20 a	78.0 ± 12.0 a	31.5 ± 24.7 ab
Rootstock	101-14M	0.56 ± 0.07 a	0.72 ± 0.14 a	62.9 ± 10.1 ab	49.7 ± 14.4 a
	110R	0.34 ± 0.10 d	0.43 ± 0.16 cd	53.3 ± 12.3 bc	17.5 ± 16.6 cd
	188-08	0.38 ± 0.12 bcd	0.35 ± 0.14 d	62.2 ± 25.2 ab	59.7 ± 18.5 a
	3309C	0.45 ± 0.12 bc	0.62 ± 0.18 ab	67.5 ± 14.8 a	27.5 ± 18.2 bc
	5BB	0.36 ± 0.16 cd	0.52 ± 0.13 bc	47.0 ± 25.1 c	5.6 ± 7.6 d
	5C	0.43 ± 0.12 abc	0.69 ± 0.12 a	60.6 ± 22.3 ab	18.8 ± 16.7 cd
	SO_4_	0.45 ± 0.21 bc	0.61 ± 0.18 ab	70.8 ± 16.9 a	13.3 ± 12.3 cd
	Beta	0.47 ± 0.13 ab	0.54 ± 0.08 bc	70.0 ± 7.4 a	45.0 ± 22.8 ab
Subject	Scion	92.3 ***	37.8 ***	69.1 ***	3.6 *
	Rootstock	12.6 ***	21.8 ***	8.2 ***	20.8 ***
	Scion × Rootstock	6.1 ***	4.3 ***	5.4 ***	2.1 *

Note: Within each column for each group of factors, different letters indicate significant differences (*p* < 0.05) according to Tukey’s post hoc test. CFI indicates callus forming index, and GHI indicates graft healing index. Within each column for between-subject effects, data are F values followed by asterisks, where * indicates *p* < 0.05 and *** indicates *p* < 0.001.

**Table 2:** Growth-related indices of three grapevine cultivars on eight rootstocks over two years.

Factor	Subfactor	Survival Rate %	Rootstock Diameter (mm)	Scion Diameter (mm)	AC	Total Cane Length (cm)	Cane Diameter (mm)
Scion	CS	93.8 ± 3.7 a	16.4 ± 2.6 a	13.5 ± 3.9 a	1.3 ± 0.2 b	214.9 ± 88.2 a	11.7 ± 0.7 b
	MS	85.2 ± 9.0 b	15.4 ± 3.5 b	13.9 ± 5.1 a	1.2 ± 0.2 c	186.1 ± 129.9 b	13.6 ± 0.9 a
	PV	86.3 ± 8.6 b	15.2 ± 3.2 b	11.8 ± 4.1 b	1.4 ± 0.3 a	168.4 ± 91.4 c	12.0 ± 0.6 b
Rootstock	101-14M	91.9 ± 7.5	15.8 ± 3.2 ab	13.0 ± 4.3	1.3 ± 0.2	200.8 ± 107.7 a	12.4 ± 0.7
	110R	88.9 ± 9.8	15.7 ± 3.1 ab	12.5 ± 4.3	1.4 ± 0.3	195.5 ± 104.3 ab	12.3 ± 1.1
	188-08	91.0 ± 6.7	15.5 ± 3.6 ab	13.1 ± 4.4	1.3 ± 0.2	190.8 ± 108.5 ab	12.3 ± 0.6
	3309C	87.7 ± 8.9	15.3 ± 3.0 b	13.0 ± 4.2	1.3 ± 0.2	209.2 ± 126.9 a	12.1 ± 1.3
	5BB	85.8 ± 10.3	16.5 ± 3.4 a	13.6 ± 5.1	1.3 ± 0.3	189.6 ± 105.7 ab	12.8 ± 0.9
	5C	87.1 ± 9.6	15.8 ± 3.0 ab	12.9 ± 4.5	1.3 ± 0.3	197.4 ± 118.0 a	12.5 ± 1.1
	SO_4_	91.7 ± 9.4	15.6 ± 3.3 ab	13.4 ± 5.1	1.3 ± 0.3	187.5 ± 112.3 ab	12.3 ± 1.0
	Beta	85.1 ± 6.2	14.9 ± 3.0 b	12.6 ± 4.3	1.3 ± 0.2	162.3 ± 88.5 b	12.7 ± 1.8
	Own root	86.5 ± 5.6	-	13.5 ± 4.6	-	175.3 ± 94.0 ab	12.6 ± 1.6
Year	2018	88.4 ± 8.3	12.9 ± 1.4	9.0 ± 1.2	1.5 ± 0.2	96.6 ± 39.7	-
	2019	0.0 ± 0.0	18.4 ± 1.7	17.1 ± 2.2	1.1 ± 0.1	286.6 ± 58.4	12.4 ± 1.1
Subject	Scion	9.9 ***	17.8 ****	61.3 ****	50.2 ****	27.6 ****	59.8 ****
	Rootstock	1.0	3.2 **	2.4 *	2.7 *	3.3 **	0.9
	Year	-	1050.6 ****	2382.6 ****	586.8 ****	1296.1 ****	-
	Scion × Rootstock	0.7	5.1 ****	5.3 ****	3.4 ***	3.2 ***	1.9 *
	Scion × Year	-	21.9 ****	27.6 ****	2.0	46.7 ****	-
	Rootstock × Year	-	1.5	2.5 *	2.2 *	3.3 **	-
	Scion × Rootstock × Year	-	2.2 *	2.1 *	1.6	1.7 *	-

Note: Within each column for each group of factors, different letters indicate significant differences (*p* < 0.05) according to Tukey’s post hoc test. AC indicates the affinity coefficient. Within each column for between-subject effects, data are F values followed by asterisks, where * indicates *p* < 0.05, ** indicates *p* < 0.01, *** indicates *p* < 0.001, and **** indicates *p* < 0.0001.

**Table 3:** Petiole nutrients in three grapevine cultivars on eight rootstocks at florescence and veraison stages across two years.

Factor	Subfactor	N (mg g^−1^)	P (mg g^−1^)	K (mg g^−1^)	Ca (mg g^−1^)	Mg (mg g^−1^)	S (mg g^−1^)	B (mg kg^−1^)	Cu (mg kg^−1^)	Fe (mg kg^−1^)	Mn (mg kg^−1^)	Zn (mg kg^−1^)
Scion	CS	13.4 ± 6.1 a	4.9 ± 2.4 a	36.1 ± 7.6 a	27.5 ± 8.7 a	10.7 ± 3.4 a	7.5 ± 3.5 a	28.6 ± 3.5 c	23.8 ± 17.4 b	43.2 ± 17.5 b	176.8 ± 175.2 b	39.8 ± 11.4 b
	MS	11.8 ± 5.5 b	3.4 ± 2.0 b	23.3 ± 12.4 c	22.6 ± 5.9 b	9.5 ± 3.3 b	5.8 ± 2.3 b	30.4 ± 3.5 b	21.0 ± 16.6 b	48.2 ± 33.2 a	313.3 ± 198.7 a	32.7 ± 10.9 c
	PV	13.2 ± 4.8 a	3.5 ± 1.9 b	31.0 ± 6.8 b	23.4 ± 7.0 b	9.1 ± 3.1 b	7.7 ± 2.7 a	33.6 ± 5.0 a	33.9 ± 26.8 a	50.5 ± 18.6 a	318.4 ± 262.8 a	52.0 ± 14.6 a
Rootstock	101-14M	13.8 ± 6.8 ab	3.0 ± 1.7 b	32.0 ± 12.3 ab	22.7 ± 6.6 b	10.3 ± 3.7 ab	8.1 ± 3.6 a	32.7 ± 5.2 a	27.5 ± 21.3	43.5 ± 17.5	220.3 ± 146.1 cd	39.7 ± 10.9 bc
	110R	12.9 ± 5.8 bc	2.9 ± 1.6 b	31.4 ± 9.1 ab	26.1 ± 8.2 a	9.6 ± 3.4 abc	7.8 ± 2.9 abc	31.1 ± 5.4 abc	25.3 ± 20.7	47.0 ± 21.6	239.6 ± 167.1 cd	36.9 ± 12.9 c
	188-08	13.3 ± 5.4 abc	4.6 ± 2.8 a	33.6 ± 12.3 a	19.9 ± 4.9 c	9.2 ± 3.3 c	7.9 ± 3.4 ab	31.8 ± 4.6 abc	28.3 ± 28.4	46.9 ± 19.6	164.3 ± 122.3 d	38.0 ± 13.9 c
	3309C	13.1 ± 5.5 bc	4.3 ± 2.4 a	31.7 ± 9.7 ab	25.9 ± 7.6 a	9.4 ± 3.0 bc	7.1 ± 2.2 a–d	30.8 ± 5.3 bc	28.7 ± 23.8	50.6 ± 21.1	261.9 ± 253.0 bc	41.3 ± 14.8 bc
	5BB	14.3 ± 6.0 a	4.2 ± 2.2 a	29.0 ± 9.6 bc	27.6 ± 7.2 a	9.7 ± 3.3 abc	6.4 ± 2.4 cd	28.8 ± 3.5 d	24.2 ± 19.7	44.3 ± 15.1	324.5 ± 242.9 b	44.8 ± 15.0 ab
	5C	12.6 ± 5.5 cd	4.0 ± 2.3 a	25.8 ± 9.7 c	27.7 ± 7.3 a	10.1 ± 3.4 abc	6.9 ± 3.3 a–d	30.3 ± 4.3 bcd	25.3 ± 19.9	44.1 ± 17.5	203.2 ± 139.6 cd	40.9 ± 12.8 bc
	SO_4_	12.7 ± 5.4 bcd	4.2 ± 2.2 a	28.7 ± 8.6 bc	25.8 ± 7.4 a	9.4 ± 3.0 bc	6.2 ± 2.4 d	30.7 ± 3.9 bc	26.5 ± 21.5	47.0 ± 21.4	283.6 ± 256.6 bc	44.2 ± 15.9 ab
	Beta	11.7 ± 4.2 de	4.6 ± 2.1 a	34.5 ± 11.8 a	18.7 ± 5.9 c	9.4 ± 4.2 bc	6.2 ± 2.8 d	30.0 ± 3.6 cd	24.8 ± 18.1	47.4 ± 17.0	495.5 ± 308.3 a	48.8 ± 18.9 a
	Own root	11.2 ± 4.5 e	3.9 ± 1.7 a	25.6 ± 8.3 c	26.1 ± 7.2 a	10.6 ± 2.8 a	6.5 ± 3.3 bcd	31.9 ± 4.2 ab	26.3 ± 21.0	53.6 ± 47.9	241.1 ± 165.1 cd	40.5 ± 14.9 bc
Stage	florescence	17.2 ± 4.5 a	5.5 ± 1.5 a	32.7 ± 9.4 a	19.7 ± 4.4 b	7.1 ± 2.0 b	7.3 ± 2.1	33.6 ± 4.3 a	8.7 ± 1.8 b	43.6 ± 15.3 a	125.4 ± 93.8 b	38.1 ± 13.2 b
	veraison	8.4 ± 1.3 b	2.4 ± 1.6 b	27.7 ± 11.1 b	29.4 ± 6.9 a	12.4 ± 2.1 a	6.8 ± 3.7	28.2 ± 3.0 b	44.1 ± 17.3 a	51.1 ± 30.1 b	414.6 ± 226.0 a	45.2 ± 15.5 a
Year	2020	11.0 ± 3.2 b	4.0 ± 2.1	33.4 ± 11.0 a	24.0 ± 8.9 b	9.1 ± 4.1 b	8.2 ± 3.5 a	31.2 ± 4.0	28.6 ± 24.4 a	60.0 ± 27.5 a	221.0 ± 195.6 b	34.8 ± 9.4 b
	2021	14.5 ± 6.6 a	4.0 ± 2.3	27.3 ± 9.3 b	25.0 ± 6.1 a	10.3 ± 2.3 a	5.9 ± 1.9 b	30.6 ± 5.0	24.3 ± 18.4 b	35.4 ± 10.5 b	314.1 ± 241.5 a	47.9 ± 16.1 a
Subject	Scion	40.2 ****	73.4 ****	140.5 ****	57.5 ****	35.9 ****	26.0 ****	109.7 ****	52.7 ****	7.9 ***	61.5 ****	167.0 ****
	Rootstock	12.4 ****	12.4 ****	11.1 ****	31.0 ****	4.9 ***	4.1 ***	7.4 ****	0.8	1.6	25.9 ****	6.9 ****
	Stage	2768.0 ****	693.9 ****	75.8 ****	571.0 ****	1177.4 ****	3.7	337.7 ****	1144.8 ****	27.3 ****	550.1 ****	56.8 ****
	Year	465.6 ****	0.0	79.4 ****	10.4 **	66.0 ****	89.0 ****	3.1	17.5 ***	223.4 ****	50.0 ****	229.9 ****
	Stage × Scion	10.4 ***	1.4	81.5 ****	31.6 ****	7.5 ***	21.9 ****	22.8 ****	31.4 ****	14.4 ****	15.2 ****	1.6
	Stage × Rootstock	7.8 ****	2.0 *	6.9 ****	4.4 ***	4.3 ***	4.0 ***	3.9 ***	0.8	3.8 ***	9.2 ****	5.3 ****
	Stage × Year	446.2 ****	53.7 ****	56.9 ****	38.4 ****	169.3 ****	49.7 ****	13.7 ***	19.8 ***	32.6 ****	0.0	8.9 **
	Scion × Rootstock	1.3	6.9 ****	2.4 **	1.2	1.7	2.2 **	2.1 *	1.3	3.8 ****	1.8 *	2.7 ***
	Scion × Year	24.3 ****	6.1 **	7.1 **	22.3 ****	30.3 ****	5.2 **	9.1 ***	2.4	7.7 ***	2.7	17.1 ****
	Rootstock × Year	0.6	1.0	0.4	0.5	0.6	1.8	0.6	0.5	1.6	0.6	2.7 **
	Stage × Scion × Rootstock	2.4 **	0.8	0.5	1.3	0.7	0.9	1.1	1.2	3.2 ***	1.2	2.3 **
	Stage × Scion × Year	7.0 **	2.7	2.1	16.4 ****	2.7	28.0 ****	5.9 **	2.3	8.3 ***	2.2	1.3
	Stage × Rootstock × Year	1.0	0.5	0.4	0.7	1.4	0.7	0.8	0.8	3.3 **	0.4	0.7
	Scion × Rootstock × Year	0.8	1.8 *	1.1	1.1	0.8	1.3	1.0	1.9 *	3.2 ***	0.4	2.4 **
	Stage × Scion × Rootstock× Year	0.7	0.6	0.6	1.2	1.2	0.3	0.8	2.2 **	3.4 ***	0.5	1.1

Note: Within each column for each group of factors, different letters indicate significant differences (*p* < 0.05) according to Tukey’s post hoc test. Within each column for between-subject effects, data are F values followed by asterisks, where * indicates *p* < 0.05,** indicates *p* < 0.01, *** indicates *p* < 0.001, and **** indicates *p* < 0.0001.

## Data Availability

The datasets generated during this study are fully available within the article.

## Supplementary Materials

The following supporting information can be downloaded at: https://www.mdpi.com/article/10.3390/plants15182797/s1, Figure S1: Heatmaps of factor effect size ($\eta_{p}^{2}$) on (a) graft compatibility, (b) growth indicators, and (c) petiole mineral levels; Figure S2: Heatmaps of petiole mineral levels of three grape cultivars on eight rootstocks at two phenological stages across two years. CS, ‘Cabernet Sauvignon’; MS, ‘Marselan, PV, ‘Petit Verdot’; Figure S3: One-way ANOVA of petiole mineral nutrients among combinations of three grape cultivars on eight rootstocks at the florescence stage in 2020. CS, ‘Cabernet Sauvignon’; MS, ‘Marselan, PV, ‘Petit Verdot’; Figure S4: One-way ANOVA of petiole mineral nutrients among combinations of three grape cultivars on eight rootstocks at the veraison stage in 2020. CS, ‘Cabernet Sauvignon’; MS, ‘Marselan, PV, ‘Petit Verdot’; Figure S5: One-way ANOVA of petiole mineral nutrients among combinations of three grape cultivars on eight rootstocks at the florescence stage in 2021. CS, ‘Cabernet Sauvignon’; MS, ‘Marselan, PV, ‘Petit Verdot’; Figure S6: One-way ANOVA of petiole mineral nutrients among combinations of three grape cultivars on eight rootstocks at the veraison stage in 2021. CS, ‘Cabernet Sauvignon’; MS, ‘Marselan, PV, ‘Petit Verdot’; Figure S7: Linear regression analysis of the graft healing index (GHI) against the callus forming index (CFI). CS, ‘Cabernet Sauvignon’; MS, ‘Marselan, PV, ‘Petit Verdot’; Figure S8: Linear regression analysis of petiole mineral concentrations in own-rooted scion cultivars against their respective overall means. CS, ‘Cabernet Sauvignon’; MS, ‘Marselan, PV, ‘Petit Verdot’; Table S1: Details of scion and rootstock cultivars; Table S2: Summary of the meteorological data; Table S3: One-way ANOVA of grafting-related indices; Tabel S4: One-way ANOVA of growth-related indices among graft combinations of three scions and eight rootstocks across two years; Table S5: Correlation coefficient and *p*-value matrices among grafting coefficients, growth performance, and petiole mineral levels at two developmental stages; Table S6: Multi-way ANOVA of petiole mineral contents among own-rooted ‘Cabernet Sauvignon’ (CS), ‘Marselan’ (MS) and ‘Petit Verdot’ (PV) at two stages across two years.

## Abbreviations

The following abbreviations are used in this manuscript:

CS	Cabernet Sauvignon
MS	Marselan
PV	Petit Verdot
NAA	naphthylacetic acid
IAA	indole-3-acetic acid
CFI	callus-forming index
GHI	graft-healing index
VSP	vertical shoot positioning
AC	affinity coefficient
ICP-OES	Inductively Coupled Plasma-Optical Emission Spectrometry
ANOVA	analysis of variance
PCA	principal component analysis.
