# Supplementary material for: Scion Influence and Rootstock Specificity in Grafted Grapevines: Insights into Graft Affinity, Growth, and the Dynamics of Nutrient Acquisition
Source: Plants (Basel). 2026 Sep 12;15(18):2797. doi: 10.3390/plants15182797 (PMC13610937; doi:10.3390/plants15182797)
Supplement: Supplementary file 1 [file plants-15-02797-s001.zip › Supplementary Figures.pdf]

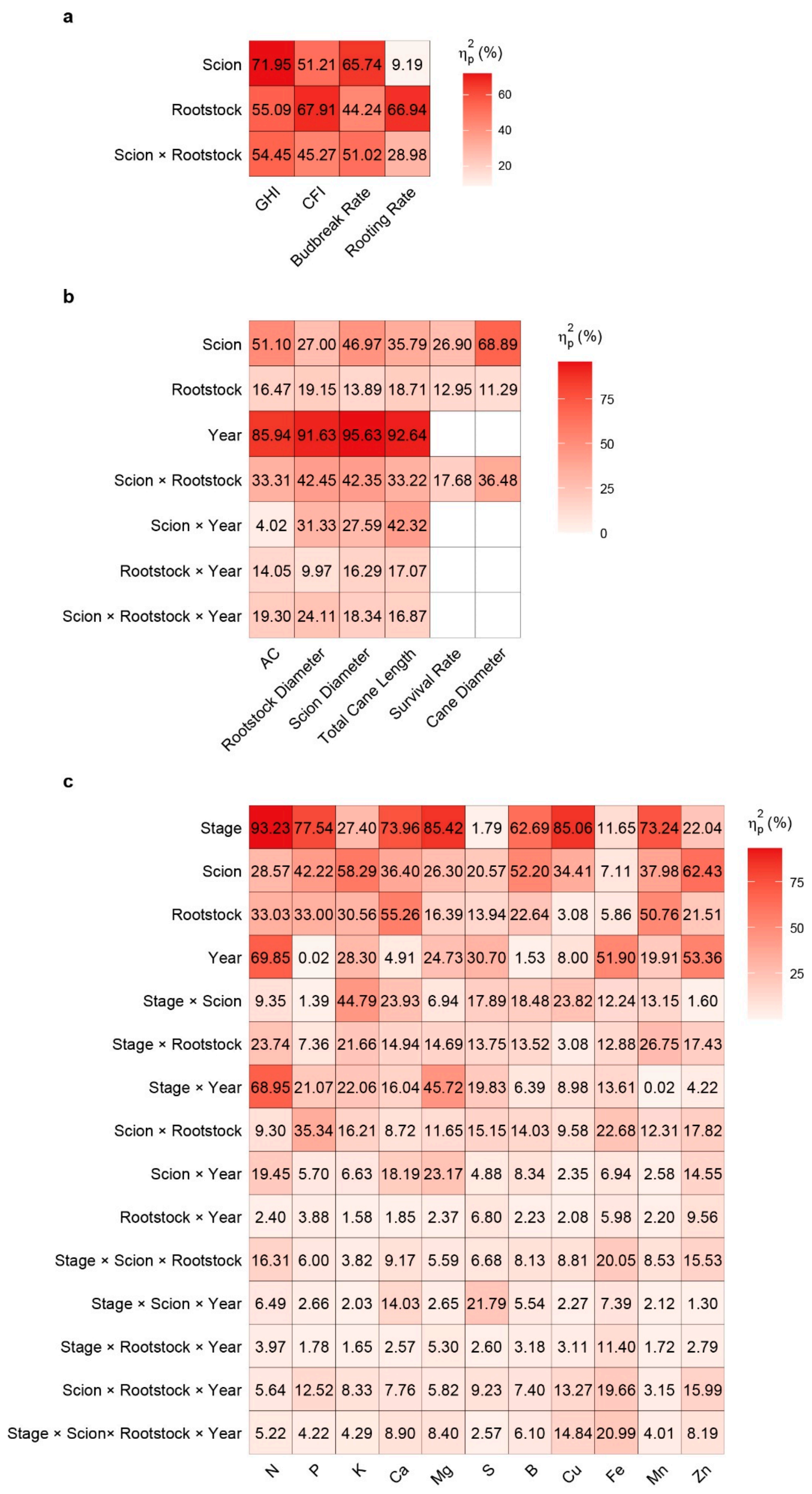

Figure S1. Heatmaps of factor effect size (η_p^2) on (a) graft compatibility, (b) growth indicators, and (c) petiole mineral levels.

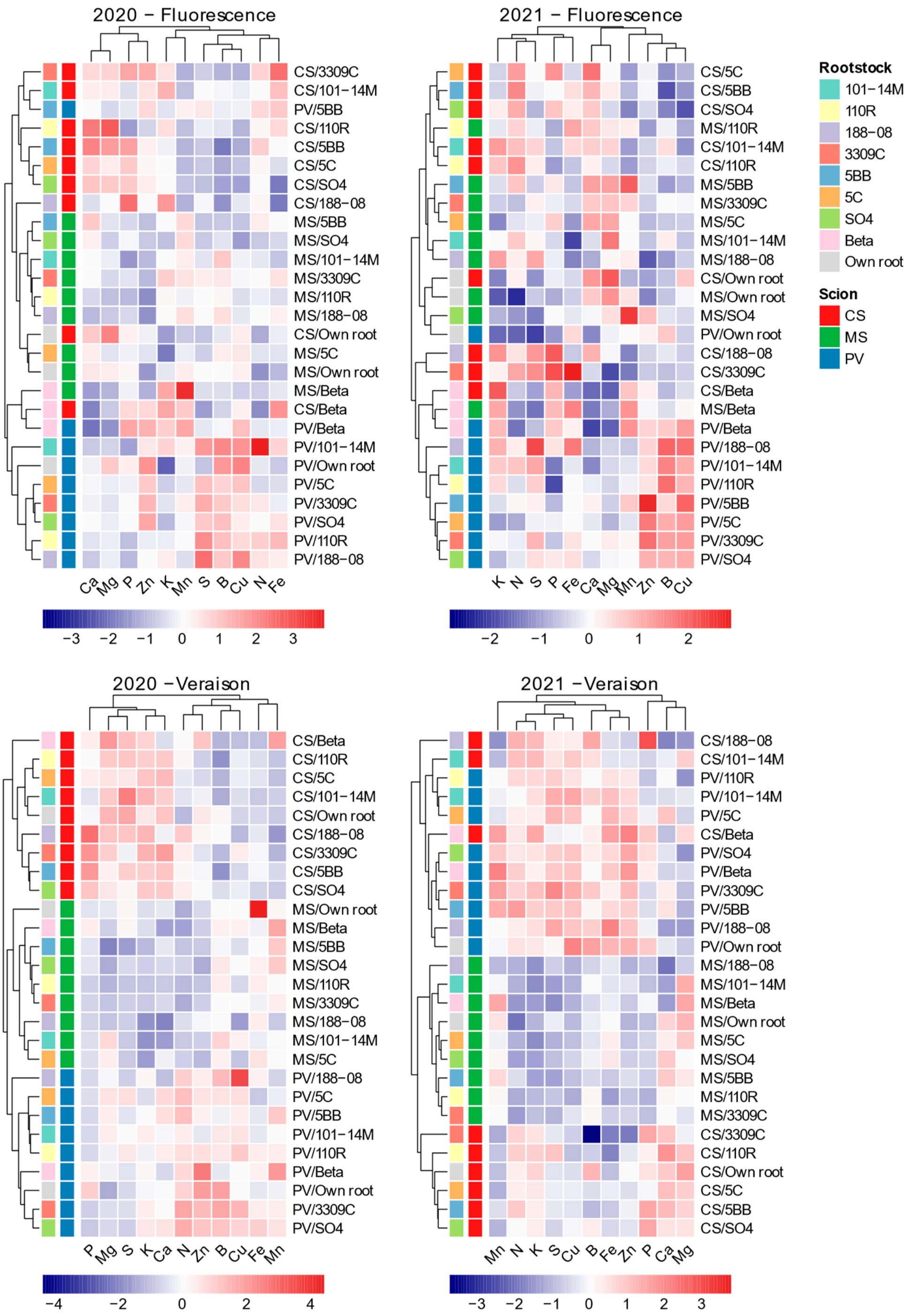

Figure S2. Heatmaps of petiole mineral levels of three grape cultivars on eight rootstocks at two phenological stages across two years. CS, 'Cabernet Sauvignon'; MS, 'Marselan, PV, 'Petit Verdot'.

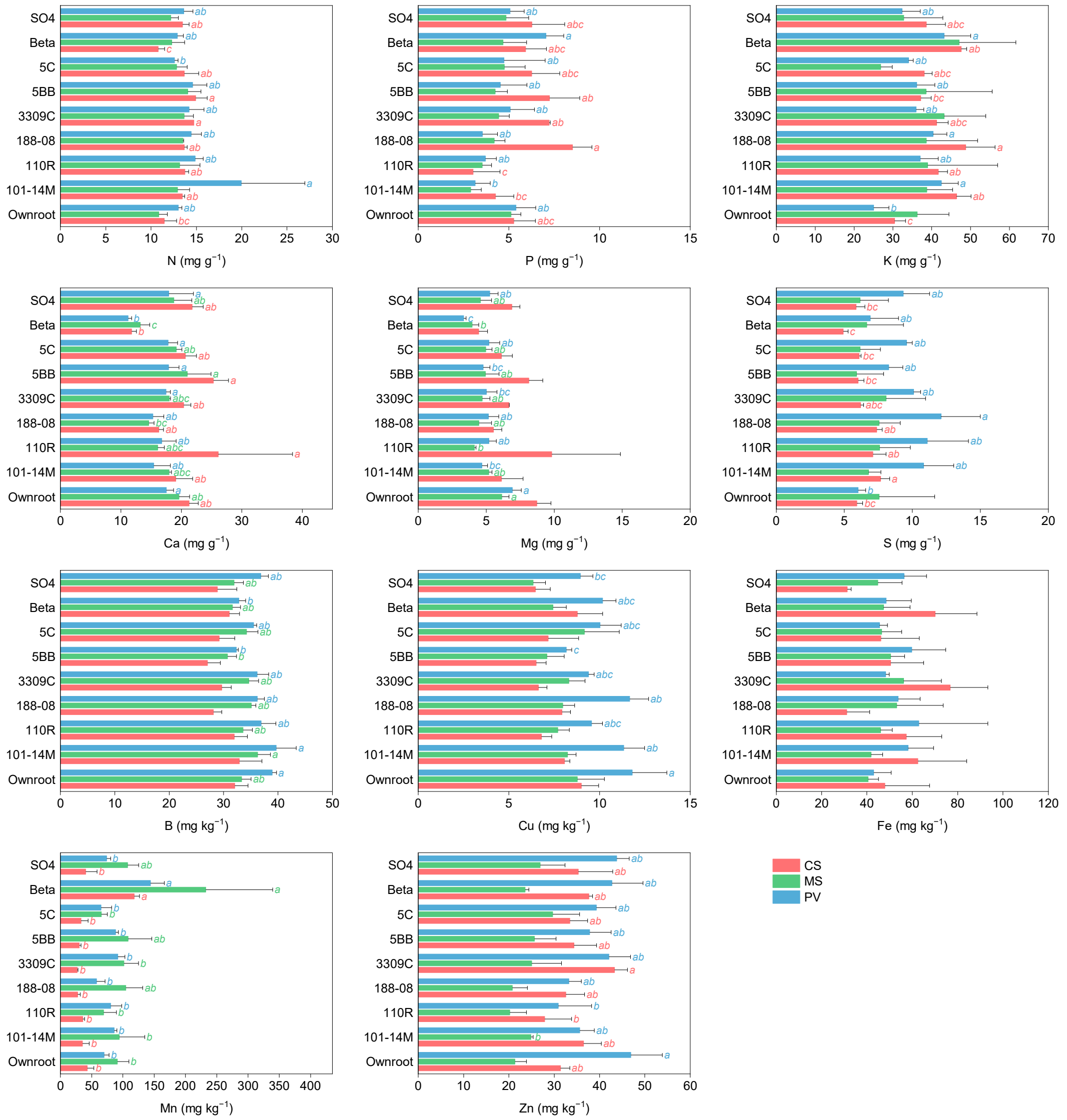

Figure S3. One-way ANOVA of petiole mineral nutrients among combinations of three grape cultivars on eight rootstocks at the florescence stage in 2020. CS, 'Cabernet Sauvignon'; MS, 'Marselan, PV, 'Petit Verdot'.

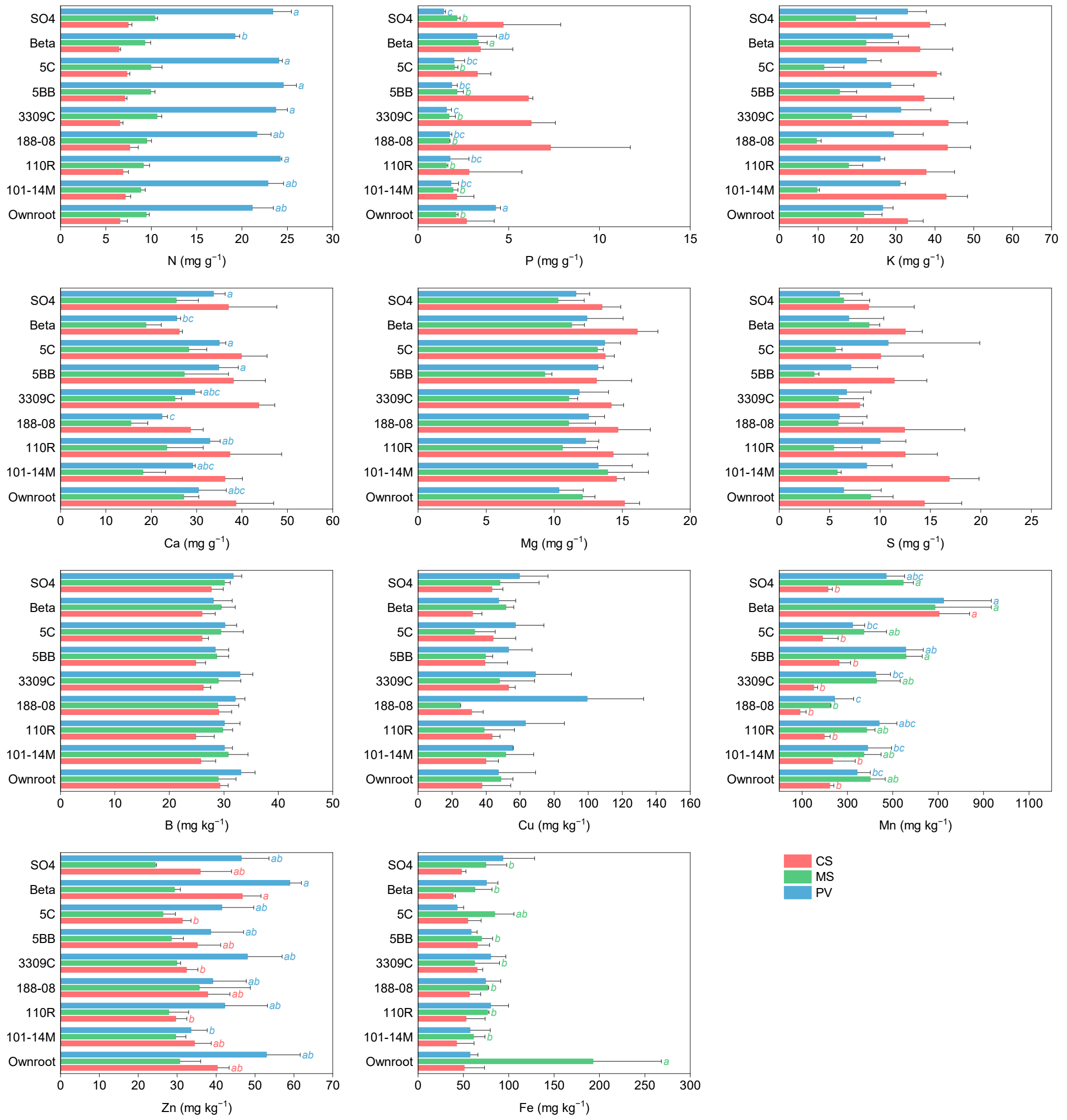

Figure S4. One-way ANOVA of petiole mineral nutrients among combinations of three grape cultivars on eight rootstocks at the veraison stage in 2020. CS, 'Cabernet Sauvignon'; MS, 'Marselan, PV, 'Petit Verdot'.

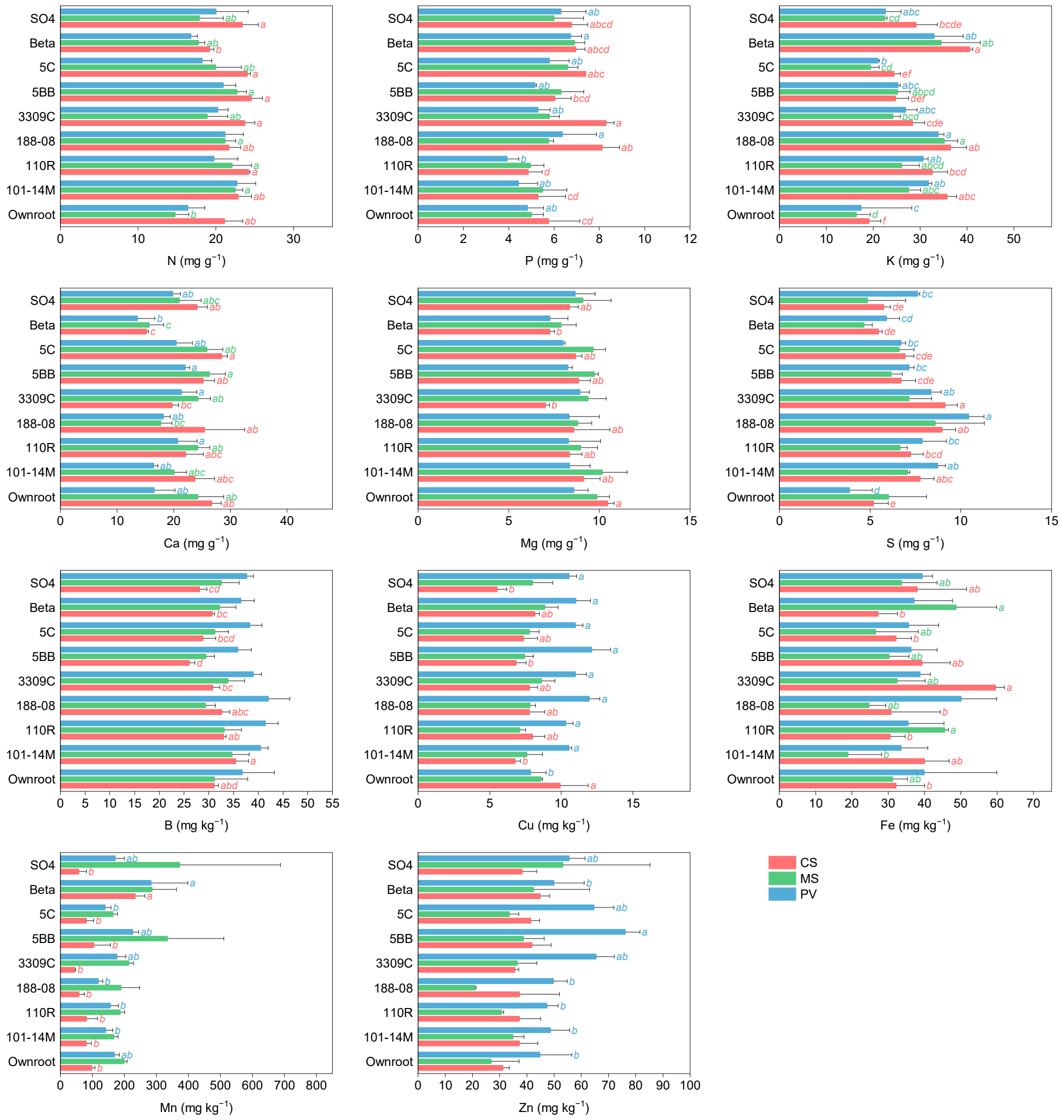

Figure S5. One-way ANOVA of petiole mineral nutrients among combinations of three grape cultivars on eight rootstocks at the florescence stage in 2021. CS, 'Cabernet Sauvignon'; MS, 'Marselan, PV, 'Petit Verdot'.

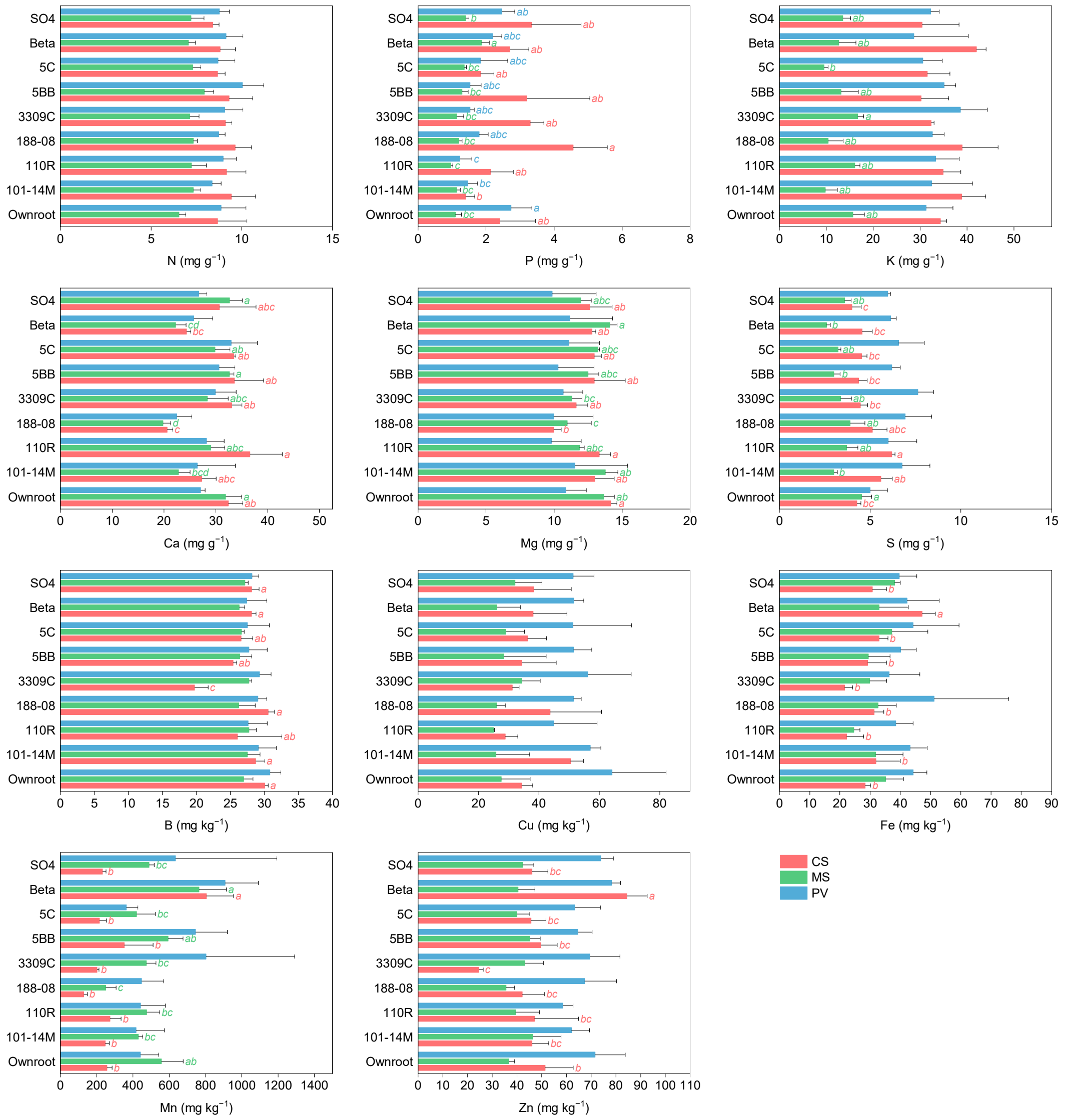

Figure S6. One-way ANOVA of petiole mineral nutrients among combinations of three grape cultivars on eight rootstocks at the veraison stage in 2021. CS, 'Cabernet Sauvignon'; MS, 'Marselan, PV, 'Petit Verdot'.

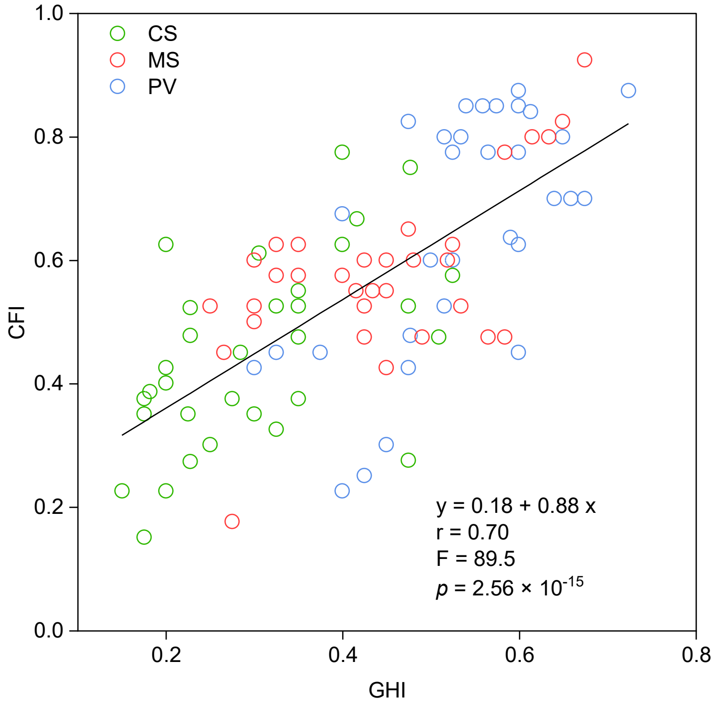

Figure S7. Linear regression analysis of the graft healing index (GHI) against the callus forming index (CFI). CS, 'Cabernet Sauvignon'; MS, 'Marselan, PV, 'Petit Verdot'.

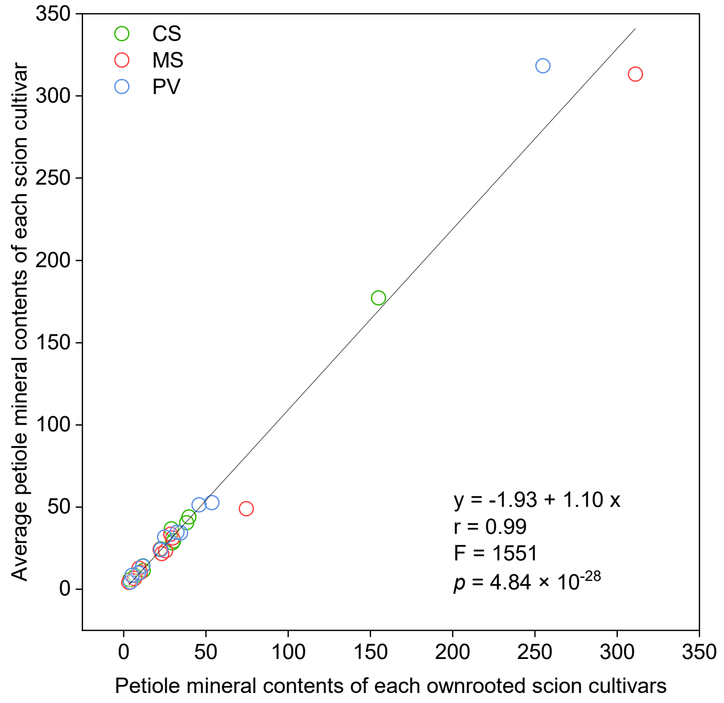

Figure S8. Linear regression analysis of petiole mineral concentrations in own-rooted scion cultivars against their respective overall means. CS, 'Cabernet Sauvignon'; MS, 'Marselan, PV, 'Petit Verdot'.
